# Phospholipid membranes drive abdominal aortic aneurysm development through stimulating coagulation factor activity

**DOI:** 10.1073/pnas.1814409116

**Published:** 2019-04-03

**Authors:** Keith Allen-Redpath, Maceler Aldrovandi, Sarah N. Lauder, Anastasia Gketsopoulou, Victoria J. Tyrrell, David A. Slatter, Robert Andrews, W. John Watkins, Georgia Atkinson, Eileen McNeill, Anna Gilfedder, Majd Protty, James Burston, Sam R. C. Johnson, Patricia R. S. Rodrigues, Dylan O. Jones, Regent Lee, Ashok Handa, Keith Channon, Samya Obaji, Jorge Alvarez-Jarreta, Gerhard Krönke, Jochen Ackermann, P. Vince Jenkins, Peter W. Collins, Valerie B. O’Donnell

**Affiliations:** ^a^Systems Immunity Research Institute, School of Medicine, Cardiff University, CF14 4XN Cardiff, United Kingdom;; ^b^Division of Infection and Immunity, School of Medicine, Cardiff University, CF14 4XN Cardiff, United Kingdom;; ^c^Division of Cardiovascular Medicine, British Heart Foundation Centre for Research Excellence, Radcliffe Department of Medicine, University of Oxford, OX3 9DU Oxford, United Kingdom;; ^d^Nuffield Department of Surgical Sciences, University of Oxford, OX3 9DU Oxford, United Kingdom;; ^e^Department of Internal Medicine, University Hospital Erlangen, 91054 Erlangen, Germany;; ^f^Institute for Clinical Immunology, University Hospital Erlangen, 91054 Erlangen, Germany;; ^g^Haematology Department, University Hospital of Wales, CF14 4XW Cardiff, United Kingdom

**Keywords:** aneurysm, lipid, phospholipid, lipoxygenase, angiotensin

## Abstract

Abdominal aortic aneurysm (AAA) is an inflammatory vascular disease with high mortality and limited treatment options. How blood lipids regulate AAA development is unknown. Here lipidomics and genetic models demonstrate a central role for procoagulant enzymatically oxidized phospholipids (eoxPL) in regulating AAA. Specifically, through activating coagulation, eoxPL either promoted or inhibited AAA depending on tissue localization. Ang II administration to *ApoE*^*−/−*^ mice increased intravascular coagulation during AAA development. Lipidomics revealed large numbers of eoxPL formed within mouse and human AAA lesions. Deletion of eoxPL-generating enzymes (*Alox12* or *Alox15*) or administration of the factor Xa inhibitor rivaroxaban significantly reduced AAA. *Alox*-deficient mice displayed constitutively dysregulated hemostasis, including a consumptive coagulopathy, characterized by compensatory increase in prothrombotic aminophospholipids (aPL) in circulating cell membranes. Intravenously administered procoagulant PL caused clotting factor activation and depletion, induced a bleeding defect, and significantly reduced AAA development. These data suggest that *Alox* deletion reduces AAA through diverting coagulation away from the vessel wall due to eoxPL deficiency, instead activating clotting factor consumption and depletion in the circulation. In mouse whole blood, ∼44 eoxPL molecular species formed within minutes of clot initiation. These were significantly elevated with *ApoE*^*−/−*^ deletion, and many were absent in *Alox*^*−/−*^ mice, identifying specific eoxPL that modulate AAA. Correlation networks demonstrated eoxPL belonged to subfamilies defined by oxylipin composition. Thus, procoagulant PL regulate AAA development through complex interactions with clotting factors. Modulation of the delicate balance between bleeding and thrombosis within either the vessel wall or circulation was revealed that can either drive or prevent disease development.

## Significance

Abdominal aortic aneurysm (AAA) is a disease of the abdominal aorta where inflammation causes damage and can ultimately lead to rupture. When this happens, uncontrolled internal bleeding can lead to death within minutes. Many aneurysms are not detected until they rupture, and for those that are, treatments to stop them progressing are limited. Here we used biophysics and genetically modified mice to show that a new family of lipids (fats) made by circulating blood cells promote AAA formation in the vessel wall because they directly regulate blood clotting. An approach that prevents AAA development was identified, based on intravenous administration of lipids. The studies provide insights into how AAA develops and may lead to novel therapies for this disease.

Abdominal aortic aneurysms (AAA) form in the abdominal aorta and cause ∼11,000 deaths per year in the United Kingdom and United States, due to sudden rupture (1, 2). There are limited options to alter the natural history of AAA development. Treatment usually requires surgery; however, these operations are associated with high rates of morbidity and mortality. A recent metaanalysis indicated that genetic control of lipoprotein levels alters the risk of developing AAA; however, the role of bioactive inflammatory lipids is unknown (3).

Coagulation factor activation has been observed in human AAA, and a potentially causative role in disease is suggested because some anticoagulant drugs reduce AAA formation; however, the mechanisms that underpin this are unknown (4–7). Lipids play an important role in controlling blood clot formation. For hemostasis to occur, coagulation factors must assemble on phospholipid (PL) membranes leading to thrombin generation. Two classes of PL work together to achieve coagulation, aminoPL (aPL) and enzymatically oxidized PL (eoxPL). aPL comprise phosphatidylethanolamine (PE) and phosphatidylserine (PS), and both are absent on the outer surface of resting blood cells. During injury, blood cells including platelets externalize aPL, generating an electronegative surface. The PS headgroup associates with calcium allowing binding of coagulation factors (factors II, VII, IX, and X) to the cell surface, increasing their local concentrations and allowing them to interact (8–11). PS is essential, and its procoagulant activity is enhanced by PE (12, 13). A key role for aPL in supporting in vivo blood clotting on the surface of eosinophils was demonstrated recently (14).

In the last 10 years, large families of oxidized PL generated by circulating immune cells, macrophages, and platelets were discovered. These are called eoxPL since they are generated in a controlled manner by immune cell enzymes (15–21). eoxPL are required for normal hemostasis in vivo and are elevated in human venous thrombosis (14, 17, 22). In isolated platelets, over 100 molecular species were recently identified using lipidomics, and additional forms are generated by neutrophils and monocytic cells (20). eoxPL are formed by lipoxygenases (LOX), of which there are several immune cell isoforms. These are encoded by *ALOX15/Alox15* (human 15-LOX1, murine 12/15-LOX in eosinophils, resident peritoneal macrophages, and IL4-treated monocytes), *ALOX12/Alox12* (12-LOX, platelets) and *ALOX5/Alox5* (5-LOX, neutrophils). There are also rarer isomers generated by cyclooxygenase-1 (COX-1, *PTGS/Ptgs1*) (15).

We recently elucidated the detailed biochemical mechanisms by which eoxPL promote coagulation (17, 21, 22). On cell activation, these lipids are externalized to the outer plasma membrane where they interact with clotting factors (21, 23). Using multiple approaches, coagulation factors were shown to bind eoxPL membranes, directly enhancing catalytic turnover (22). Their electronegative oxidized fatty acid side chains enhance the ability of PS to support clotting (22). When administered directly at a site of injury, eoxPL support hemostasis in wild-type or hemophilia A mice through provision of a local surface for coagulation factor binding and activation, where it is needed (17, 22). We also recently found that *Alox12*^−/−^ and *Alox15*^−/−^ mice generate smaller venous thrombi and bleed excessively when challenged and that hemostasis can be restored by local eoxPL injection into damaged tissue (14, 17). However, it is not yet known which eoxPL molecular species form during clot formation, the predominant forms contributing to hemostasis/thrombosis, or how their interactions with coagulation may influence vascular inflammation.

Herein, we hypothesize that eoxPL regulation of coagulation may play a role in AAA development. To test this, we focused on characterizing the generation and role of the procoagulant surface provided by eoxPL in angiotensin II-driven disease, using genetic murine models, oxylipidomics mapping of whole blood, and analysis of human AAA tissue.

## Results

### Lipidomic Profiling Demonstrates Multiple eoxPL Species in Developing AAA Lesions.

**Fig. 1.**
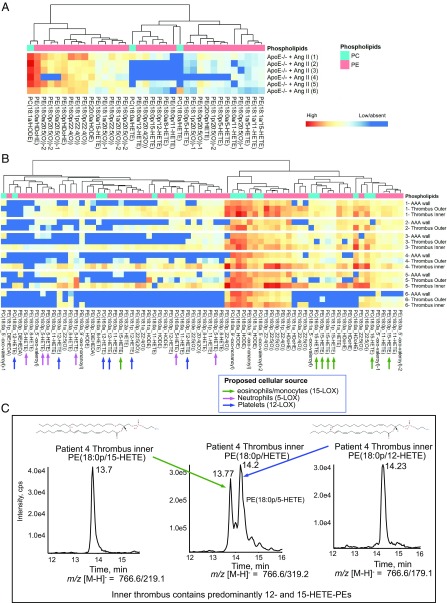
EoxPL are detected in *ApoE*^−/−^ mouse and human AAA tissue. (*A*) Lipidomics shows eoxPL in murine AAA. Lipids from abdominal aorta harvested from *ApoE*^*−/−*^ mice following Ang II was analyzed using LC/MS/MS, as in *SI Appendix*, *SI Materials and Methods* (each sample represents a different aorta). (*B*) Lipidomics reveals eoxPL in human AAA. Abdominal aorta were harvested from six male patients, and lipids were analyzed using LC/MS/MS. Heatmaps were generated using Pheatmap as described in *SI Appendix*, *SI Materials and Methods* (each sample represents a different sample), shown as log10 values for analyte:internal standard normalized to tissue weight (mg). (*C*) Representative chromatograms show 18:0p/HETE-PE lipids, with a predominance of eoxPL from either Alox15 or Alox12. *Middle* shows *m*/*z* 766.6/319.2, detecting all HETE-PE isomers and revealing two main products. These are confirmed as 15-HETE-PE (13.7 min) or 12-HETE-PE (14.2 min), by detecting internal daughter ions at *m*/*z* 219.1 or 179.1, respectively.

To examine for the generation of procoagulant PL in AAA lesions, molecular species of eoxPL were profiled using targeted LC/MS/MS in aortic tissue from 19- to 24-wk-old chow-fed male *ApoE*^*−/−*^ mice administered 1.1 mg⋅kg^−1^ per day of Ang II by osmotic minipump for 2 wk. A large number of eoxPL were detected, including hydroxyoctodecadienoic acid (HODE); hydroxydocosahexanoic acid (HDOHE); and 5-, 11-, 12-, and 15-hydroxyeicosatetraenoic acid (HETE)-PL, including both phosphatidylethanolamines (PE) and phosphatidylcholines (PC) (Fig. 1*A*). In contrast, eoxPL were undetectable in aortic wall from *ApoE*^*−/−*^ mice not administered Ang II.

**Fig. 2.**
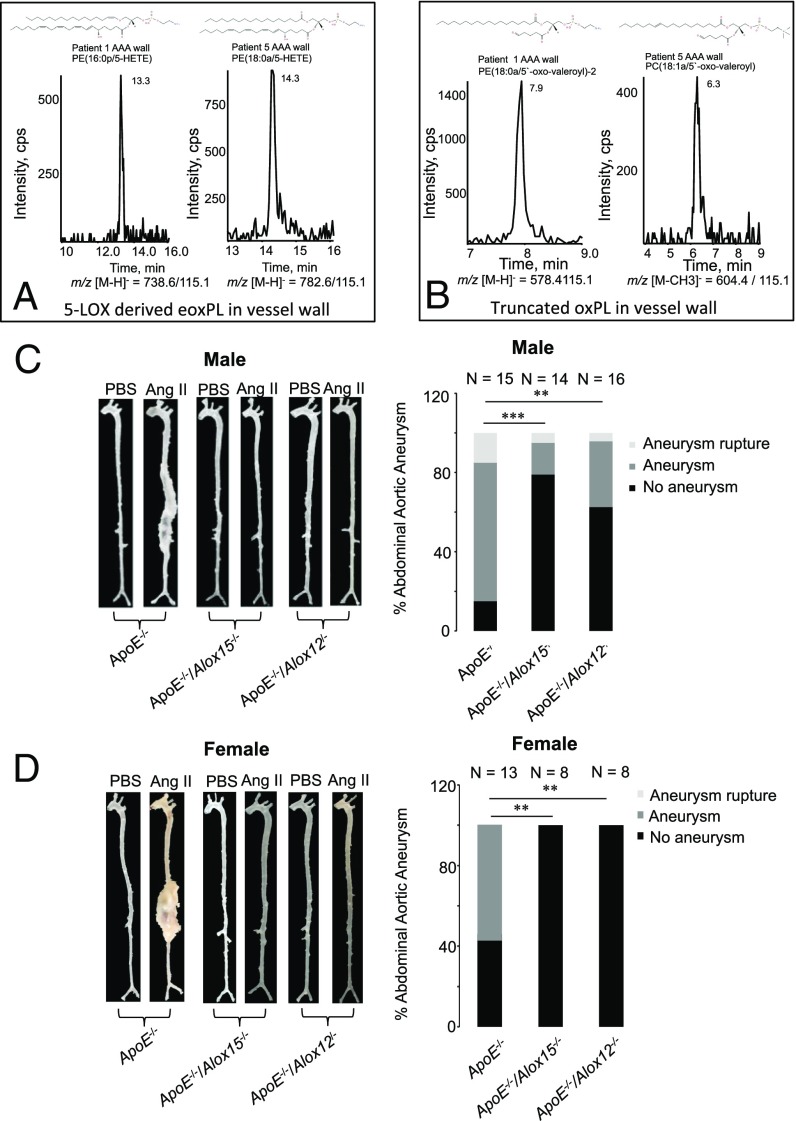
The 12- and 15-LOX–derived eoxPL are detected in human AAA thrombus, while Alox12 or Alox15 deletion prevents AAA. (*A*) Representative chromatograms showing a truncated PE and PC both detected in AAA wall. (*B*) Representative chromatograms of eoxPL generated by leukocyte 5-LOX, in AAA wall. (*C* and *D*) *Alox15* or *Alox12* deletion reduces AAA development in *ApoE*^*−/−*^ mice. Male (*C*) or female (*D*) *ApoE*^*−/−*^, *ApoE*^*−/−*^/*Alox15*^*−/−*^, and *ApoE*^*−/−*^/*Alox12*^*−/−*^ mice aged 19–24 wk were infused with Ang II for 2 wk. Aortae were imaged as described in *Materials and Methods*. Representative aortae are shown (*Left*), including a PBS control for each condition. The full datasets are provided in *SI Appendix*, Figs. S3–S5. Incidence of AAA as percent of group is shown (*Right*). Data are analyzed using Fisher’s exact test and expressed as percent AAA, ***P < 0.001, **P < 0.01.

In a preliminary analysis, we examined for eoxPL generation in human AAA and its mural thrombus. Here six patient samples were divided into aortic wall, inner thrombus (closest to lumen), and outer thrombus (closest to aneurysm wall) (Figs. 1 *B* and *C* and 2 *A* and *B* and *SI Appendix*, Fig. S1). Despite AAA patients varying due to age, demographics, and genetic background, there were clear similarities between donors, with the same lipid species generally predominating. For all samples, eoxPL were detected in thrombus and AAA wall, with a trend toward higher levels and greater diversity in the thrombi. Focusing on HETE-PLs, molecular species containing 12- or 5-HETEs were primarily detected in thrombus (from 12-, 5-LOXs in platelets and neutrophils), while 15-HETE-PLs (from 15-LOX in eosinophils and monocytes) were found in all locations (Fig. 1*B*). As a representative dataset, inner thrombus from patient 4 contains three HETE-PE molecular species with each comprising two abundant isomers containing 12- and 15-HETE (Fig. 1*C* and *SI Appendix*, Fig. S1). Representative chromatograms are shown for two 5-HETE-PEs for patients 1 and 5 (Fig. 2*A*). OxPL containing truncated PUFA were found in AAA wall and thrombi (Figs. 1*B* and 2*B*). Truncated species may form via nonenzymatic fragmentation of enzymatically generated full-length eoxPL. This preliminary analysis, which supports the idea that eoxPL are a component of human AAA, now needs to be repeated using larger numbers of AAA samples, enabling deeper characterization and correlation with clinical stage, medication, inflammatory markers, gene expression, etc.

### AAA Development Is Significantly Reduced in *ApoE*^−/−^ Mice Lacking Either *Alox12* or *Alox15*.

Since eoxPL were detected in AAA tissue, we tested whether genetic deletion of two *Alox* isoforms that generate this class of lipids in isolated blood cells affects lesion development (18, 21, 24). We specifically focused on *Alox12* (platelets, 12-LOX) and *Alox15* (leukocytes, 12/15-LOX). *ApoE*^*−/−*^ mice were backcrossed, generating *ApoE*^−/−^/*Alox12*^−/−^ and *ApoE*^−/−^/*Alox15*^−/−^ double knockout strains. These were first confirmed to be resistant to atherosclerosis development, as described in *SI Appendix*, Fig. S2. Next, to examine the effect of *Alox*-deletion on AAA, Ang II was administered to male or female *ApoE*^−/−^, *ApoE*^−/−^/*Alox12*^−/−^, or *ApoE*^−/−^/*Alox15*^−/−^ mice for 2 wk, as above. For both *Alox*-deficient strains, AAA development was markedly reduced in male and absent in female mice (Fig. 2 *C* and *D* and *SI Appendix*, Figs. S3–S5). This demonstrates that both isoforms can independently contribute to disease development. This protection was independent of blood pressure (BP) and plasma lipoprotein levels since a significant BP elevation was seen in all strains at day 11, while there was no effect of *Alox* deletion on total cholesterol with/without Ang II treatment (*SI Appendix*, Fig. S6*A* and Tables S1 and S2). Overall, the disease burden in females was lower, in line with previous studies; thus, protection against AAA was more complete in that gender (25).

### Direct Inhibition of FXa Significantly Reduced AAA Development in Vivo.

An involvement of coagulation in AAA development has been suggested (7), and we recently demonstrated that eoxPL, specifically HETE-PEs and HETE-PCs, bind to and support clotting factor activities (17, 22). To test whether AAA is dependent on coagulation factor activity in this model, we administered the direct FXa inhibitor rivaroxaban via chow to male *ApoE*^−/−^ mice during Ang II administration and found significantly less AAA developed (*SI Appendix*, Fig. S6*B*). This suggested a mechanism by which *Alox15* and *Alox12* could promote AAA development, where eoxPL generated at the vessel wall during lesion progression provide a procoagulant surface for clotting factor binding and activation. This idea was next investigated by characterizing coagulation activity in the *Alox*-deficient mouse strains basally and during Ang II infusion.

### Alox12 or Alox15 Deficiency Disrupts Coagulation in Wild-Type or ApoE^−/−^ Mice.

**Fig. 3.**
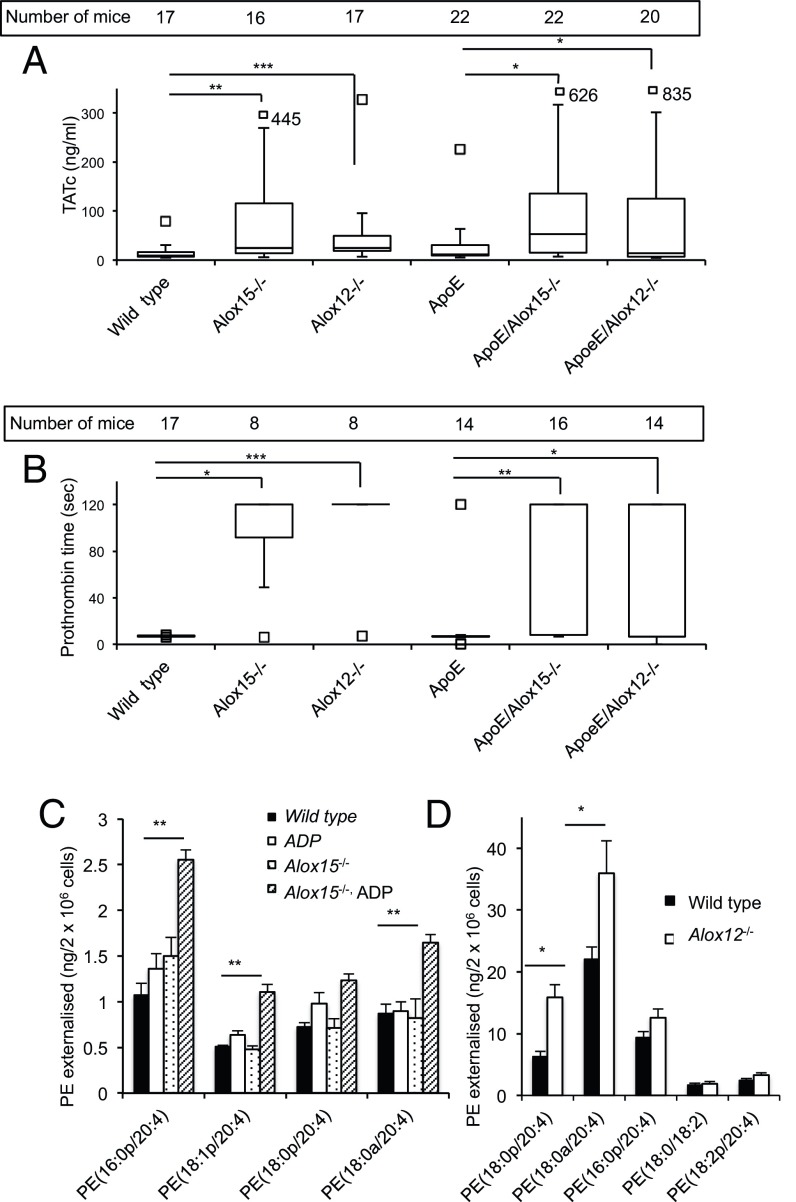
*Alox12* ^−/−^ or *Alox15* ^−/−^ mice show disruption of the thrombosis/bleeding axis, with increased aPL externalization in circulating blood cells. (*A*) *Alox12* or *Alox15* deficiency promotes thrombin generation in vivo in wild-type and *ApoE*^*−/−*^ mice. Plasma from 19-wk-old mice was isolated as in *Materials and Methods*. Thrombin/anti-thrombin (TAT) complexes were measured using ELISA. (*B*) *Alox12* or *Alox15* deficiency reduces clotting in wild-type and *ApoE*^*−/−*^ mice. Mouse plasma obtained as in *B* was tested for PT as in *Materials and Methods*. For *A* and *B*, data were analyzed using Mann–Whitney nonparametric *u* test and shown on box plots (median, with whiskers representing interquartile range), ***P < 0.001, **P < 0.01, *P < 0.05. (*C*) Eosinophils from *Alox15*^*−/−*^ mice externalize more PE than wild type on ADP activation. Eosinophils generated as described in *Materials and Methods* were activated using 40 μM ADP before PE externalization determined (*n* = 3, mean ± SEM). (*D*) Platelets from *Alox12*^*−/−*^ mice externalize more PE than wild type on thrombin activation. Mouse platelets were isolated as described in *Materials and Methods*, and PE externalization was determined. Data were analyzed using Student’s *t* test and expressed as mean ± SEM (*n* = 10). **P < 0.01, *P < 0.05 (*n* = 10).

Circulating thrombin-anti-thrombin (TAT) complexes were increased approximately twofold in *ApoE*^*−/−*^ versus wild type, although this was not significant (Fig. 3*A*). However, in either wild-type or *ApoE*^−/−^ mice, genetic deletion of either *Alox12* or *Alox15* led to approximately fivefold elevations in TATs (Fig. 3*A*), indicating significantly higher levels of thrombin generation in *Alox*-deficient mice. We next measured prothrombin time (PT) following addition of tissue factor-containing PL to platelet poor plasma (PPP). This is sensitive to levels of clotting factors II, VII, IX and X, and increases with relative factor deficiency, with a value of 120 s reflecting severe coagulopathy. Overall, PT significantly increased in both wild-type and *ApoE*^−/−^ mice following deletion of *Alox12* or *Alox15* (Fig. 3*B*). Both TAT and PT values in *Alox*^*−/−*^ single or double knockouts showed far higher variability than either wild-type or *ApoE*^*−/−*^ mice, indicating that the extent of *Alox*^*−/−*^-associated coagulopathy varies between individual mice. Also, the effect of *Alox* deletion was not affected by *ApoE* deficiency. These findings demonstrate an intravascular consumptive coagulopathy. Since this occurs in the absence of vascular inflammatory challenge, it indicates that *Alox12*- or *Alox15*-deficient mice exhibit chronic defective coagulation under basal conditions. Potential explanations for this finding were sought, as given below. There was a higher variation of both PT and TAT in both *Alox*-deficient strains. We believe this is due to variable penetrance of the phenotype, a not uncommon feature of genetically modified mouse strains (26–28). The biological reasons in these strains are unknown.

### Deficiency of *Alox12*^−/−^ or *Alox15*^−/−^ Increases PE Externalization on the Surface of Platelets or Eosinophils.

Consumptive coagulopathy in *Alox12*^−/−^ and *Alox15*^−/−^ mice was initially unexpected since these mice are deficient in procoagulant eoxPL lipids (17, 24). However, we note that depletion of coagulation factors could at least in part explain the protective AAA phenotype since the model is FXa dependent (*SI Appendix*, Fig. S6*B*). To explore mechanisms, we examined exposure of procoagulant aPL on the surface of washed platelets or eosinophils (derived from bone marrow progenitors) from *Alox12*^−/−^ or *Alox15*^−/−^ mice, respectively. *Alox15*^−/−^ eosinophils externalized significantly more PE on ADP activation (Fig. 3*C*), while *Alox12*^−/−^ platelets externalized significantly more PE without stimulation (Fig. 3*D*). These data suggest that deficiency of eoxPL is overcompensated for by elevation in aPL externalization in circulating cells. This is likely to lead to activation of coagulation, increased thrombin generation and depletion of clotting factors within the circulation, as was indeed observed in these mice.

### Coagulation Is Activated During AAA Development and Modulated by Genetic Deficiency of Alox Isoforms.

Given the requirement for coagulation in driving Ang II-dependent AAA and the basal clotting defects in *Alox*^−/−^ mice, the effect of Ang II infusion on systemic coagulation and how this is modulated by *Alox* deletion was next characterized.

**Fig. 4.**
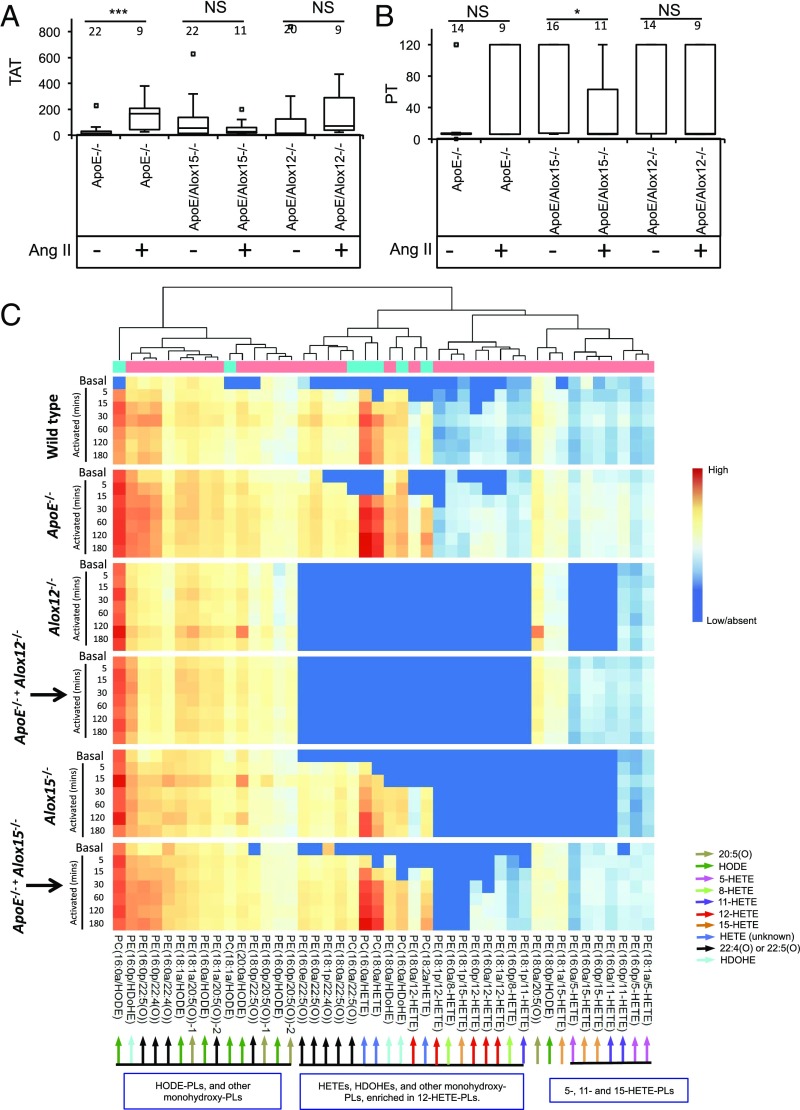
Ang II infusion induces consumptive coagulopathy in mice, while *ApoE* and *Alox* deletion is associated with major changes in eoxPL profile. (*A*) Ang II increases plasma TATs in mice. *ApoE*^−/−^, *ApoE*^−/−^/*Alox12*^−/−^, and *ApoE*^−/−^/*Alox15*^−/−^ 19- to 24-wk-old male mice maintained on a normal chow diet were infused with Ang II for 2 wk. TATs were measured by ELISA. (*B*) PT of plasma in response to 2-wk infusion of Ang II with/without liposomes. *ApoE*^−/−^, *ApoE*^−/−^/*Alox12*^−/−^, and *ApoE*^−/−^/*Alox15*^−/−^ 19- to 24-wk-old male mice maintained on a normal chow diet were administered Ang II for 2 wk. PT was measured as per *Materials and Methods*. Data were analyzed using Mann–Whitney nonparametric *u* test and shown on box plots (median, with whiskers representing interquartile range), ***P < 0.001, *P < 0.05. (*C*) Oxylipidomics of blood clotting from all mouse strains. Blood was harvested and induced to clot as in *Materials and Methods*. Lipids were extracted from clots and profiled using LC/MS/MS. Heatmaps were generated using Pheatmap (*n* = 3 per sample) using log10 data values as described in *SI Appendix*, *SI Materials and Methods*.

First, we found that Ang II infusion mediated significant increases in plasma TATs in *ApoE*^−/−^ mice, along with more variable TAT levels in this group (Fig. 4*A*). While PT did not increase significantly, wide variability in PT was also seen following Ang II infusion (Fig. 4*B*). Indeed, several mice (5 out of 9) showed the maximum of 120 s (assay termination), versus only 1 of 14 in the control *ApoE*^*−/−*^ group. Together, this indicates that Ang II stimulates a consumptive coagulopathy during AAA development in the majority of mice. Given the protective effect of rivaroxaban and localization of eoxPL (Fig. 1*A* and *SI Appendix*, Fig. S6*B*), coagulation factors are likely activated at the vessel wall and may drive lesion development through so far uncharacterized inflammatory mechanisms.

In contrast, *ApoE*^−/−^/*Alox15*^−/−^ and *ApoE*^−/−^/*Alox12*^−/−^ mice showed no significant changes in TATs following Ang II treatment (Fig. 4*A*). Also, PT values were not consistently altered in *ApoE*^−/−^/*Alox15*^−/−^ or *ApoE*^−/−^/*Alox12*^−/−^ strains, respectively, by Ang II (Fig. 4*B*). However, both TATs and PT were already significantly elevated in both double knockout strains before Ang II infusion, compared with *ApoE*^*−/−*^ alone (Fig. 3 *A* and *B*). Thus, in the double knockout strains, these data may simply reflect both *ApoE*- and *Alox*-dependent coagulation taking place at the same time. Notably, while wild-type or *ApoE*^*−/−*^ mice showed a low level of variation between individuals, the extent of coagulopathy seen with either Ang II or *Alox* deficiency was highly variable between individual mice.

Overall, these data indicate that Ang II-driven AAA is associated with an acute consumptive coagulopathy that promotes lesion development. However, conversely, the chronic coagulopathy seen in *Alox* deficiency is associated with protection. Although this may seem paradoxical, these different outcomes may reflect different localizations of coagulation. Specifically, acute Ang II-driven coagulation is expected to be vessel wall localized (*SI Appendix*, Scheme S1*A*), while in *Alox* deficiency, basal activation of coagulation and factor consumption may occur on the aPL-exposing surface of circulating blood cells/platelets (*SI Appendix*, Scheme S1 *B* and *C*). This chronic activation of coagulation seen in *Alox*^*−/−*^ mice may then lead to a relative lack of factors available to locally bind and stimulate vascular inflammation in the vessel wall in response to Ang II, thus dampening AAA development in double knockout mice.

### Lipidomics Reveals Complex Regulation of eoxPL by ApoE, Alox12, and Alox15 During Coagulation.

The endogenous molecular species of eoxPL that support AAA development in vivo are unknown, although several candidates were detected in lesions (Fig. 1). To delineate which originate from *Alox12* or *Alox15* and the effect of vascular inflammation, oxylipidomics of a forming murine thrombus comparing all six murine strains was performed. Since *Alox*^*−/−*^ mice are protected against AAA, it is not possible to obtain sufficient lesional tissue from double knockout mice; thus, blood was used as a surrogate tissue. Murine whole blood was induced to clot by TF in vitro, modeling a low-shear venous-type thrombus, containing plasma, white cells, red cells, and platelets. In wild-type blood, 44 eoxPL including both PEs and PCs formed, peaking around 15–20 min, then declining back to baseline (Fig. 4*C*). In contrast, *ApoE*^−/−^ blood responded in an exaggerated manner, generating higher levels of eoxPL that remained elevated for longer.

In contrast, clots from *Alox12*^*−/−*^ or *Alox15*^*−/−*^ mice lacked large numbers of eoxPL, in particular several HETEs, HDOHEs, and monohydroxy forms of adrenic (22:4) and eicosapentanoic (22:5) acids, and these remained low when mice were backcrossed to *ApoE*^−/−^ (Fig. 4*C*). Notably, these tend to be longer-chain PUFA, which are classical substrates for *Alox* isoforms. The same lipids were largely reduced in both strains, and this is expected since 12-LOX and 12/15-LOX display almost identical enzymatic activities. A group of lipids that were enriched in 5- and 15-HETEs, most likely from neutrophils in mice (e.g., from *Alox5* or *Ptgs1*), was reduced in *Alox12*^−/−^ or *Alox15*^−/−^ mice; however, backcrossing to *ApoE*^−/−^ restored their levels (Fig. 4*C*). This suggests a positive influence of *Alox12/Alox15* on neutrophil activation during blood clotting. Last, a family of eoxPL enriched in HODEs of unknown origin is somewhat elevated in *Alox12*^−/−^- or *Alox15*^−/−^-deficient clots (versus wild type), and these were only partially influenced by *ApoE*^−/−^ status.

Hierarchical clustering of heatmap lipids shows significant grouping based on the *Sn2* oxylipin structure (Fig. 4*C*). Comparing the pattern and abundance of eoxPL signals, we suggest that the molecular species most likely to interact with coagulation factors driving AAA in vivo are HETE-PEs (Fig. 4*C*). Thus, we next focused on measuring these isoforms in all strains during clot formation, using a targeted quantitative assay.

### HETE-PEs Are Elevated in ApoE^−/−^ During Clotting but Decrease on Backcrossing to Alox12^−/−^ or Alox15^−/−^.

HETE-PE species were quantified as a subgroup of eoxPL, using biogenic standards. *ApoE*^−/−^ clots generated twice the levels as wild type (*SI Appendix*, Fig. S7*A*). However, *Alox12*^−/−^ or *Alox15*^−/−^ deficiency, either wild type or on *ApoE*^*−/−*^ background, significantly reduced total HETE-PEs (*SI Appendix*, Fig. S7*A*). In particular, *Alox12*^*−*/−^ reduced HETE-PEs back to approximately wild-type levels in *ApoE*^−/−^ mice. These data further support the idea that HETE-PEs represent the eoxPL primarily responsible for the effect of *Alox* on AAA development.

### Multivariate Analysis Reveals eoxPL That Describe Mouse Strain Differences During Clot Formation.

Next, the full eoxPL dataset was statistically analyzed using principle component analysis in 3D (*SI Appendix*, Fig. S7*B*). Unclotted blood from all strains (t = 0 min) grouped closely (solid short arrows), indicating similar lipid composition. Once coagulation was initiated, divergence was seen, and this was strongly influenced by genotype. At the end (t = 180 min), most strains are found in separate regions of the plot indicating different lipid compositions (dashed long arrows). A loadings plot indicates that most eoxPL have a strong positive influence in PC1 (*SI Appendix*, Fig. S7*C*). Notably, headgroup did not influence loadings in either PC1 or PC2. However, fatty acyl at *Sn2* has a clear influence in PC2, with 5-, 15-HETEs, 20:5(O), and HODE generally having a positive effect (above the line), while 12-HETEs, HDOHEs, 22:4(O), and 22:5(O) had a negative effect (below the line). This indicates that longer-chain PUFA eoxPL are generated as a related group (negative in PC2), versus shorter chain more saturated (positive in PC2). We next explored this further using Cytoscape analysis, which correlates individual lipids with each other.

### EoxPL Cytoscape Analysis Reveals Subgroups of Molecular Species Based on Enzymatic Source.

Using Cytoscape, a correlation plot was generated, where nodes (circles) represent lipids, with edges (lines) representing the strength of correlation (*SI Appendix*, Fig. S7*D*). Size of node represents the number of correlations, with larger nodes meaning a higher number of edges per lipid. eoxPL were assigned to 12-LOX if absent in *Alox12*^−/−^ clots (*SI Appendix*, Fig. S7*D*). The 5-LOX (*Alox5*)-derived products were assigned by the presence of 5-HETE at *Sn2*. PE(18:1p/12-HETE) and PE(18:1p/15-HETE) were assigned as “LOX”-derived based on their absence in at least one *Alox* deficient strain. This analysis shows that lipids from *Alox12* are strongly correlated, while 5-HETE-PLs from *Alox5* cluster together in the middle. HODE-PLs form a separate group, to the left of the correlation plot. This indicates that eoxPL with the same *Sn2* fatty acid composition are regulated together. Along with the heatmap and principal component analysis (PCA), this reveals coordinated regulation of biosynthesis and metabolism and suggests that eoxPL subfamilies arise from differentially regulated cellular and enzymatic pathways during mouse blood clot formation, including the reesterification pathways that attach eicosanoids to lysophospholipids.

### Exogenous eoxPL or aPL Protect ApoE^−/−^ Mice Against Ang II-Induced AAA in Vivo and Influence Coagulation Regulation in Vivo.

We next tested whether eoxPL/aPL administration would alter the development of AAA in vivo, using HETE-PEs that are found in either platelets or eosinophils, from *Alox12* or *Alox15* in mice, respectively. Recently, acute eoxPL administration (1 h) was found to significantly elevate TATs, consistent with stimulating coagulation in vivo (22). Thus, we hypothesized that eoxPL administration over 2 wk might bind and activate circulating clotting factors, diverting coagulation from the vessel wall and potentially reducing AAA. Up to now, procoagulant liposomes have not been administered to mice long term, and their effect on hemostatic parameters is not known.

**Fig. 5.**
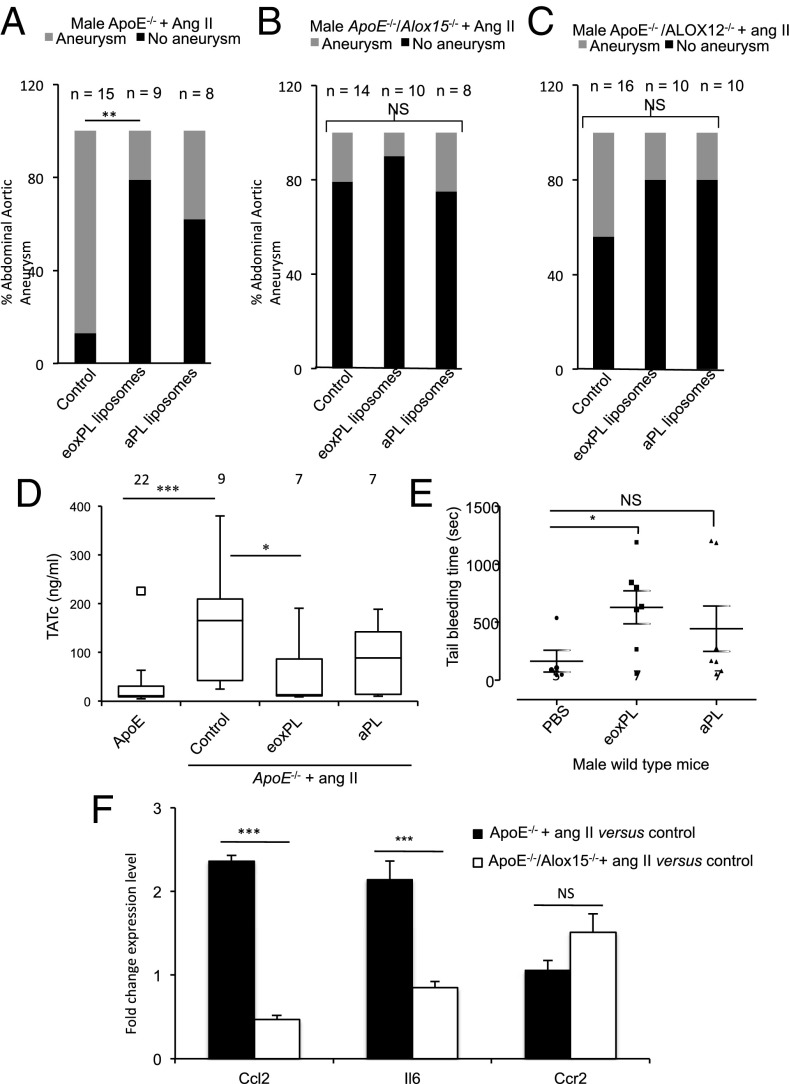
Procoagulant liposomes prevent AAA in mice, while modulating Ang II induced coagulation activities, and *Alox15* deficiency is associated with decreased Ang II induction of IL6 and Ccl2. (*A*) eoxPL/aPL liposomes prevent AAA in *ApoE*^*−/−*^ mice. Male *ApoE*^−/−^ mice on normal chow diet aged 19–24 wk were administered Ang II as described in *Materials and Methods*. Liposomes (eoxPL or aPL) were administered every second day (see *Materials and Methods* for composition and dose). Data are expressed as percentage of mice developing AAA. (*B* and *C*) eoxPL/aPL liposomes reduce AAA in *ApoE*^*−/−*^ mice lacking either *Alox12* or *Alox15*. Male mice on normal chow diet aged 19–24 wk were administered Ang II with/without liposomes as in *C*. Data are expressed as percentage of mice developing AAA. For *A*–*C*, the full dataset of aortae are shown in *SI Appendix*, Figs. S3 and S8–S10). (*D*) eoxPL liposomes alter coagulation parameters during *Ang II* infusion into *ApoE*^*−/−*^ mice. *ApoE*^−/−^ 19- to 24-wk-old male mice maintained on a normal chow diet were infused with Ang II for 2 wk, and every second day, mice eoxPL or aPL liposomes were administered as described in *Materials and Methods*. TATs were measured by ELISA. (*E*) Systemic administration of eoxPL/aPL liposomes promotes bleeding in mice. Male wild-type mice (11 wk old) were anesthetized and i.v. injected with liposomes, and tail bleeding was measured after 1 h, as described in *Materials and Methods*. Data from *A*–*C* were analyzed using Fisher’s exact test, and data from *D* and *E* were analyzed using Mann–Whitney nonparametric *u* test. ***P < 0.001, **P < 0.01, *P < 0.05; NS, no significance. Data are shown on box plots (median, with whiskers representing interquartile range). (*F*) *Alox15* deficiency reduces the Ang II-induced elevation in IL6 and Ccl2 in mouse AAA tissue. RNA was isolated from *ApoE*^*−/−*^ AAA tissues as detailed in *Materials and Methods* (*n* = 4 for all groups) and analyzed using the RT^2^ Profiler PCR Array for Mouse Inflammatory Response and Autoimmunity. Data are shown as ΔΔC_T_, expressed as fold change between control and Ang II-treated mice, and compared using Student’s *t* test. ***P < 0.005.

For this, tissue factor/phosphatidylcholine liposomes containing PS/PE (aPL liposomes), or the same liposomes with PE replaced with 12-HETE-PE (eoxPL) (10 ng per injection), were administered i.v. to male *ApoE*^−/−^ mice every second day during the Ang II infusion. In this experiment, aPL liposomes will have some procoagulant activity but significantly less than for eoxPL (22). As predicted, both formulations reduced AAA development; however, this was only statistically significant for eoxPL (Fig. 5*A*). We also administered liposomes to *ApoE*^−/−^ mice also lacking *Alox12* or *Alox15* and found a nonsignificant trend for further protection (Fig. 5 *B* and *C* and *SI Appendix*, Figs. S8–S10). Overall, this indicates that systemic provision of a procoagulant PL membrane surface reduces AAA development, with the level of protection being significantly higher with eoxPL.

Next, the effect of 2 wk eoxPL administration on plasma TAT levels was determined, during Ang II infusion. Here eoxPL or aPL administration to *ApoE*^*−/−*^ mice dampened the Ang II-dependent increase in plasma TATs, and this was statistically significant for eoxPL (Fig. 5*D*). Overall, this is in line with the idea that systemic eoxPL induce activation of coagulation in the circulation and hence divert activated clotting factors from the vessel wall, dampening the Ang II-dependent coagulopathy that is required for AAA development. While we previously found that acute eoxPL administration elevates TATs within 1 h, the overall effect on coagulation parameters of chronic eoxPL administration is not known, and further studies are required, measuring levels of individual clotting factors and their localization within the tissue compartments to delineate the underlying mechanisms responsible in this model.

### i.v. Administration of Procoagulant Liposomes Induces an Acute Bleeding Phenotype.

Our in vivo studies suggested that procoagulant lipids can be either causative (endogenous in the vessel wall) or preventative (exogenous in the systemic circulation) for AAA development (Figs. 2 and 5). To test this idea, we used hemostasis as an outcome that reports on local or systemic coagulation activity, testing the effect of eoxPL/aPL administration on tail bleeding. We showed recently that eoxPL injected locally into tail tissue prevents bleeding in wild-type mice or several strains with bleeding disorders including hemophilia A (17, 22). This is because the procoagulant surface is provided where it is needed at the cut site (17). However, in contrast, following acute i.v. injection of eoxPL into wild-type mice, tail-bleeding time significantly increased (Fig. 5*E*). Thus, eoxPL local administration promotes bleeding arrest, while systemic administration leads to a bleeding defect. Since acute i.v. eoxPL also cause TAT elevation, these data support the idea that systemic liposomes cause activation and consumption of coagulation factors (22). This is distinct from the effect of tissue-localized clotting factor activity that drives AAA in response to Ang II, despite both elevating circulating TATs. Clotting factor removal from the circulation can thus divert coagulation away from the vascular wall effectively dampening its local activities.

### Genetic Deficiency of Alox15 Suppresses Inflammation During AAA Development in Mice.

A recent study identified a central role for coagulation in driving inflammatory responses in the vessel wall during Ang II-driven disease (29). Thus, we explored whether the deletion of *Alox15* could regulate expression of *Il6* and *Ccl2/Ccr2* in aortic tissue during development of AAA. *Il6* and *Ccl2* were up-regulated during Ang II infusion in *ApoE*^−/−^ mice (Fig. 5*F*). However, this response was significantly reduced in *ApoE*^−/−^ mice lacking *Alox15*, and in the case of *Ccl2* a small decrease was noted. In contrast, *Ccr2* was relatively unaffected by Ang II in either strain (Fig. 5*F*). Overall, these data suggest that eoxPL can support the development of inflammation associated with Ang II-driven AAA, although further work is required to fully delineate the mechanisms involved and how this is mediated by clotting factors.

## Discussion

The role of the procoagulant surface provided by circulating blood cells and required for hemostasis has not been investigated in the context of AAA. Here, using genetically modified mouse models, human AAA tissue, and oxylipidomics, we demonstrate that the eoxPL/*Alox* axis is active and promotes development of AAA in wild-type mice with up-regulation of IL6/CCL2. This suggests a central role for bioactive lipids in AAA through regulating coagulation and its associated inflammation.

Murine models of AAA have been used for many years, and while they do not fully replicate the human disease, a major problem in the field is a lack of ability to follow the natural history of AAA in humans. Animal models reproduce inflammation, extracellular matrix (ECM) destruction, and aortic dilatation, all of which are seen in human aortic aneurysm (30). Similar to human disease, the Ang II/ApoE model shows preference for males and stimulates an inflammatory response, macrophage accumulation, and thrombosis (25). Here eoxPL and oxPL are detected in both murine and human AAA tissue and associated thrombus, indicating their presence at the site of disease development in both species (Figs. 1 and 2 *A* and *B*).

How thrombosis and coagulation contribute to AAA in humans is not understood; however, consumptive coagulopathy (CC) coexists with a variety of aneurysms, including presenting as disseminated intravascular coagulation (DIC) (31). This suggests pathological involvement in AAA, which our studies begin to characterize. In some patients, CC/DIC preceded and led to the diagnosis of AAA (31). In one report, a patient with stable aneurysm and chronic CC reverted to normal factor levels following repair surgery (32). Many patients experience (aneurysm-induced) DIC perioperatively (40–80%), considerably more than would be expected for routine surgery, further supporting the idea that the coagulation system is not normal in these patients. Last, focal accumulation of radiolabeled platelets in the AAA lesion in 78% of stable patients has been reported, indicating a functionally active consumption focus locally, in line with our hypothesis (33). This strongly supports the notion that factor activation in the lesion is a key feature of the disease, although identifying the exact site of thrombin generation remains a significant challenge. Indeed, thrombin generation can be sustained by isolated smooth muscle cells, in a PAR-3– or PAR-4–dependent manner, and is elevated in vitro in cells from hypertensive rats, which are known to be more susceptible to thrombosis in vivo (7, 33). The percentage of patients with DIC along with AAA is unknown since most cases are considered asymptomatic and only uncovered during the perioperative workup. Relating to inflammation, it has been estimated that around 5–10% of human AAAs are characterized by a significant inflammatory component, including an immune response, inflammatory markers, and a cuff of soft tissue inflammation surrounding the lesion (34).

Two mechanisms likely contribute to the protective effect of *Alox* deletion. First, in wild-type mice, deposition of procoagulant eoxPL within the vessel wall will support factor binding and activation locally, driving AAA. In support, Ang II infusion into *ApoE*^*−/−*^ mice is associated with factor activation, AAA is sensitive to FXa inhibition, and numerous eoxPL from both platelets and leukocytes were present in AAA lesions (Figs. 1 and 4 and *SI Appendix*, Fig. S6*B*). Furthermore, both *Alox12*^−/−^ and *Alox15*^−/−^ mice were both protected in this model, supporting a role for both platelet and leukocyte-derived eoxPL in AAA development (Fig. 2). Indeed, Ang II is already known to drive leukocyte adhesion to the vessel wall during AAA development and platelet activation (35–37). We recently found that platelet/leukocyte eoxPL promote thrombin generation in plasma (22). Monocyte/eosinophil or platelet 15- or 12-HETE-PLs are more potent at promoting thrombin generation (including ETP) than neutrophil 5-HETE-PL analogs, although all were active (22). Thus, we propose that eoxPL become deposited on the surface of the vessel wall, and these then provide a localized surface to enable coagulation factor binding and activation. The focal accumulation of radiolabeled platelets in the AAA lesion in stable human patients supports this idea (33). Last, neither *Alox12* or *Alox15* isoforms are expressed by smooth muscle or endothelium ruling these out as a potential source of eoxPL.

Second, *Alox*^*−/−*^ mice had a consumptive coagulopathy within the circulation that could reduce coagulation factor availability (Fig. 3 *A* and *B*). This is evidenced by higher TATs and intravascular depletion of clotting factors (prolonged PT), along with increases in circulating blood cell externalized aPL (Fig. 3 *C* and *D* and *SI Appendix*, Scheme S1*B*). Along with this, injection of procoagulant PL into the circulation, recently shown to acutely increase coagulation in vivo (22), caused a consumptive coagulopathy and reduced AAA incidence, similar to the protective effects of either *Alox* deletion or factor Xa inhibition (Figs. 2 and 5 and *SI Appendix*, Fig. S6*B* and Scheme S1). This provides strong evidence that diverting coagulation factors from the vessel wall prevents AAA development, highlighting the importance of tissue localization of bioactive lipids. Importantly, eoxPL/aPL can either promote or prevent AAA development, dependent on their mode of generation/delivery and site of action.

*Alox12* and *Alox15* gene products (12-LOX and 12/15-LOX) generate similar eoxPL isomers, specifically the abundant 12-HETE-PLs, and our lipidomics analysis suggests these are the most likely candidates for driving AAA in the vessel wall (Fig. 1 and *SI Appendix*, Fig. S7*A*) (18, 21, 24). This suggests potential molecular determinants that could be modified for therapeutic purposes. We note that while *Alox12*^−/−^ has a more profound effect on eoxPL levels, overall phenotypic outcome was similar with either strain. Hemostasis is a complex process during which the communication of distinct cell populations is not fully understood. Here genetic deletion of a single enzyme in platelets or myeloid cells leads to striking similarities in both health/disease phenotype and lipid mediator synthesis in blood. Along with our observation of knock-on effects of *Alox12* or *Alox15*-deletion on *Alox5* from neutrophils, this suggests that distinct blood cell types cooperate closely to orchestrate thrombosis in vivo.

We recently found that *Alox12*^−/−^ and *Alox15*^−/−^ mice generate smaller venous thrombi and bleed excessively when challenged and that hemostasis can be restored by local eoxPL injection into damaged tissue (14, 17). We proposed this resulted from deficiency in procoagulant eoxPL generated by platelets and eosinophils. Herein, we additionally show that both *Alox*^*−/−*^ strains have elevated basal TAT and PT, along with increased circulating levels of externalized aPL on blood cells (Fig. 2). This demonstrates higher endogenous rates of thrombin activation, with consumptive coagulopathy apparent in vivo under healthy conditions. This provides a second reason for a bleeding phenotype, whereby depletion of coagulation factors reduces their availability for supporting hemostasis. This is further supported by our observation that acute systemic injection of eoxPL causes consumptive coagulopathy and results in a bleeding phenotype (Fig. 5) (22).

Recently, a link between FXa generation and AAA was demonstrated in mice since enoxaparin or fondaparinux attenuated disease development, and it was suggested that hypercoagulability promotes, while inhibition of coagulation is protective (7). However, this does not take into account that thrombosis and bleeding often coexist in vascular inflammatory diseases associated with acute or chronic DIC. In DIC, hypercoagulation simultaneously leads to thrombosis and factor depletion, which presents as excess bleeding. Our data show how location of the procoagulant surface is a key determinant rather than simply the level of factors and that understanding how and where clotting factors bind and activate is critical to delineating its role in AAA development. Thus, up-regulating coagulation within the circulation by either *Alox* deletion or systemic eoxPL/aPL administration simultaneously induces a bleeding phenotype and strongly reduces AAA (14, 17, 22).

During AAA development, complex alterations to hemostasis were observed, and this was further modulated by eoxPL. We note that hemostasis will also be influenced by liver synthesis of coagulation factors and their rates of activation and clearance from the circulation, none of which are yet characterized in the context of AAA development. Furthermore, which coagulation factors are most sensitive to the actions of eoxPL in the context of AAA development are unknown. A role for FXa has been revealed; however, others may also play a role (*SI Appendix*, Fig. S6*B*) (7). A recent study revealed that blocking thrombin or FXIa could reduce hypertension and vascular remodelling in mice, raising the possibility that multiple factors mediate the effects of Ang II in vivo (29). In the case of FXI, a novel interaction with GP1bα amplified thrombin activity (29). Furthermore, blocking the interaction of GP1bα with vWF reduces thrombin generation in platelet-rich plasma (38). Given the key role of GP1bα in atherogenesis (39), further studies are required to determine the in vivo pharmacology of eoxPL effects on coagulation factor biology in AAA and their interactions with GP1bα.

How coagulation promotes AAA and the role of inflammation is not clear. A recent study found that the FXa inhibitor rivaroxaban prevents NFkB activation in a murine model of inflammation-driven cardiac remodeling (40). Here we found that genetic deletion of *Alox15* significantly abrogated the ability of Ang II to elevate both IL6 and CCL2 gene expression (Fig. 5*F*). These data support the idea that coagulation may drive inflammation in this model, in line with a recent report on the role of inflammation in Ang II-dependent cardiac remodelling (29). Future studies will characterize how blocking these pathways might affect coagulation and eoxPL generation.

Atherosclerosis is a well-known multimorbidity along with other inflammatory disorders such as chronic kidney disease, vascular dementia, diabetes, and AAA. Indeed, there is increasing interest in the underlying mechanisms by which multimorbidities develop via common and distinct pathways. Despite this, AAA does not normally develop in *ApoE*^−/−^ but requires an additional challenge (Ang II), beyond that which triggers atherosclerosis. Thus, additional pathways are required, and we show here a key involvement of eoxPL and coagulation. Given the already known role of *Alox15* in atherosclerosis, our findings underscore the central role that eoxPL and *Alox* isoforms play in several related forms of vascular inflammation.

In summary, we present a paradigm for AAA development, proposing that procoagulant cell membranes from blood cells are a driver through supporting coagulation factor activation and inflammation. The precise mechanisms by which factors such as FX (and other procoagulant and anticoagulant factors) regulate AAA along with eoxPL remain to be determined, and the role of inflammatory signaling mediated by factors requires clarification. This research highlights pathways implicated in AAA and suggests directions for future therapeutic research in this area.

## Materials and Methods

### Human Tissue Processing.

Subjects undergoing open AAA repair were prospectively recruited from the Oxford Abdominal Aortic Aneurysm (OxAAA) study, approved by the Oxford regional ethics committee (Ethics Reference: 13/SC/0250). Every participant gave written informed consent prior to the procedure. Full details are in *SI Appendix*, *SI Materials and Methods*.

### Murine Strains and Licenses.

*Alox12*^−/−^ and *Alox15*^−/−^ mice were crossed onto an *ApoE*^−/−^ strain on a C56BL/6 background. All animal experiments were performed in accordance with the United Kingdom Home Office Animals (Scientific Procedures) Act of 1986, under License (PPL 30/3150). Housing and genotyping is in *SI Appendix*, *SI Materials and Methods*.

### Atherosclerosis Quantification.

Mice were fed standard chow diet and killed via CO_2_ inhalation at 19 wk of age. Soft tissue samples were harvested for histological or immunohistochemical analysis. Samples were fixed in 4% paraformaldehyde for 48 h, which preserved tissue structure and protein expression for sectioning. Full details are in *SI Appendix*, *SI Materials and Methods*.

### Ang II Infusion, AAA Development, and Blood Pressure Recordings.

Male and female ApoE^−/−^, ApoE^−/−^/*Alox12*^−/−^, and ApoE^−/−^/*Alox15*^*−*/−^ mice (19–24 wk old) were anesthetized by inhalation with 5% isoflurane and maintained with 2% isoflurane. Osmotic minipumps (model 1002; Charles River) delivering saline or Ang II (1 mg⋅kg^−1^ per day; Sigma-Aldrich) for 14 d were implanted s.c. in the midscapular region under aseptic conditions. Full details are in *SI Appendix*, *SI Materials and Methods*.

### RNA Extraction, cDNA Synthesis, and Real-Time PCR.

AAA tissues (weight range 50–150 mg) were harvested and snap frozen. They were homogenized, and RNA was extracted as detailed in *SI Appendix*, *SI Materials and Methods*.

### PL Liposome Preparation.

Liposomes were made by extrusion in PBS, pH 7.4. Control liposomes (aPL) contained 25.67 µg DSPC, 11.52 µg SAPE, and 2.08 µg SAPS, and for eoxPL liposomes, 3.92 µg 12-HETE-PE was added with a reduced amount of SAPE (7.68 µg). Full details are in *SI Appendix*, *SI Materials and Methods*.

### Blood Lipid Analysis and Clotting Parameters.

Whole blood was collected in 3.8% sodium citrate buffer via cardiac puncture and allowed to clot for 1 h undisturbed at room temperature in a 1.5 mL Eppendorf. The blood was then centrifuged at 2,000 × *g* for 10 min at 4 °C, and the serum was removed and stored at −80 °C, before being shipped to Medical Research Council Harwell Clinical Pathology for lipid analysis. Blood was analyzed for plasma thrombin/antithrombin (TAT) and prothrombin time (PT) as outlined in *SI Appendix*, *SI Materials and Methods*.

### Isolation and Activation of Mouse Platelets.

Mouse platelets were isolated as described (17). Whole blood was obtained by cardiac puncture directly into a syringe containing 150 μL of ACD [2.5% (wt/vol) trisodium citrate, 1.5% (wt/vol) citric acid, and 100 mM glucose]. Activation is described in full in *SI Appendix*, *SI Materials and Methods*.

### Isolation and Activation of Mouse Eosinophils.

Eosinophils were generated from bone marrow isolated from 8-wk-old mice as previously described (41) with minor modifications, and activation is described in *SI Appendix*, *SI Materials and Methods*.

### Externalization of PE or PS on the Surface of Platelets and Eosinophils.

Total and external PE/PS labeled as described in full in *SI Appendix*, *SI Materials and Methods* (42). In brief, cultured mouse eosinophils (4 × 10^6^ per ml) were stimulated with ADP (40 µM) while platelets were measured basally. Full details are provided in *SI Appendix*, *SI Materials and Methods*.

### Tail Bleeding Assay.

All mice were kept in constant temperature cages (20–22 °C) and given free access to water and standard chow. Mice were anesthetized using 5% isoflurane and maintained with 2% isoflurane, and tail bleeding was measured as outlined in *SI Appendix*, *SI Materials and Methods*.

### Clot Formation Using Mouse Blood.

To model physiological clot formation, whole mouse blood was anticoagulated using citrate and corn trypsin inhibitor to prevent the contact pathway. Coagulation was initiated by recalcification at 37 °C for up to 3 h, and the use of glassware was avoided at all times, before the clot was harvested for lipid extraction and analysis, as outlined in *SI Appendix*, *SI Materials and Methods*.

### Harvesting and Processing of AAA Lesions for Lipidomics.

Male *ApoE*^−/−^ ice (18–19 wk old) were killed via CO_2_ inhalation after 2-wk Ang II infusion as outlined above. Blood was removed via cardiac puncture, and mice were perfused with PBS containing 10 mM butylatedhydroxytoluene (BHT) and diethylenetriaminepentaacetic acid (DTPA) to reduce autooxidation. Mice were carefully dissected to reveal the abdominal aorta, which was recovered and snap frozen in liquid nitrogen. Samples were stored at −80 °C until lipid extraction, as described for clots.

### Heatmap and Cytoscape Correlation.

For generation of heatmaps, analyte:internal standard for each eoxPL was plotted using the Pheatmap package in R, as described in *SI Appendix*, *SI Materials and Methods*. Relationships between related lipids were visualized in Cytoscape (version 3.6.0) using Pearson correlations generated with R (*r* > 0.8).

### Statistical Analysis.

Multivariate analysis was performed using SIMCA-P version 12.0 (Umetrics) to evaluate relationships in terms of similarity or dissimilarity among groups. PCA visualization was performed in SIMCA-P and the 3D visualization rgl package in R. All other statistics and experimental design considerations are described in *SI Appendix*, *SI Materials and Methods*.

## Supplementary Material

Supplementary File
