# Supplementary material for: Phospholipid membranes drive abdominal aortic aneurysm development through stimulating coagulation factor activity
Source: Proc Natl Acad Sci U S A. 2019 Apr 3;116(16):8038–47. doi: 10.1073/pnas.1814409116 (PMC6475397; doi:10.1073/pnas.1814409116)
Supplement: Supplementary File [file pnas.1814409116.sapp.pdf]

Supplementary Data

Supplementary Methods

Materials

Corn trypsin inhibitor (CTI, recombinant), and full-length tissue factor (TF, human recombinant) were from Haematologic Technologies Inc). 1-Stearoyl-2-arachidonoyl-phosphatidylethanolamine and -phosphatidylserine (SAPE, SAPS) and 1,2-di-stearoyl-phosphatidylcholine (DSPC) were from Avanti Polar Lipids (Alabaster, Alabama, USA). HPLC grade solvents were from Thermo Fisher Scientific (Hemel Hempstead, Hertfordshire UK). All other chemicals and lipsofast membranes were from Sigma-Aldrich. HETE-PEs were generated and purified as previously described(1).

Human aneurysm procedure.

Every participant gave written informed consent prior to the procedure. Baseline characteristics of each participant were recorded. The samples were prepared in real time, in the operating theatre. The research team has a dedicated sample preparation station housed within the operating theatre. Each specimen is prepared immediately and frozen on dry ice, and transferred for storage in -80°C freezer immediately after the case. Each thrombus was divided circumferentially to obtain the inner and outer core/rind (see below). The inner section is the part closer to the blood flow lumen, whereas the outer section is the part closer to the aneurysm wall. For big volume thrombus, the inner 1cm rind and outer 1cm rind are used. For small volume thrombus, one third of the rind is used for inner and outer sections. Tissue samples were collected during open AAA repair. Prior to incision of the aortic aneurysm, a marker pen was used to denote the cross section of maximal dilatation according to visual inspection (Panel A). After incision of the aneurysm, a longitudinal strip of the aneurysm wall along the incision was then excised. Mural thrombus within the aneurysm sac was retrieved en-bloc. No patient had aortic dissection.

www.pnas.org/cgi/doi/10.1073/pnas.1814409116

The aneurysm tissue was stripped off the peri-vascular tissue and mural thrombus. The aneurysm tissue at the maximal dilatation was isolated, divided into smaller segments, and snap frozen for subsequent analysis. Segments of the mural thrombus was demarcated alone the circumference as demonstrated by Panel B. A circumferential segment of thrombus adjacent to the blood flow lumen (luminal/inner layer) was isolated. A separate circumferential segment of thrombus adjacent to the aneurysm wall (abluminal/outer layer) was also isolated. Each of the thrombus segments were divided into smaller pieces and snap frozen for subsequent analysis.

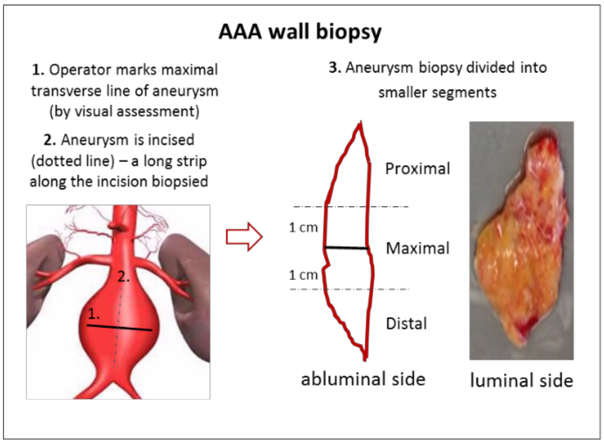

Panel A

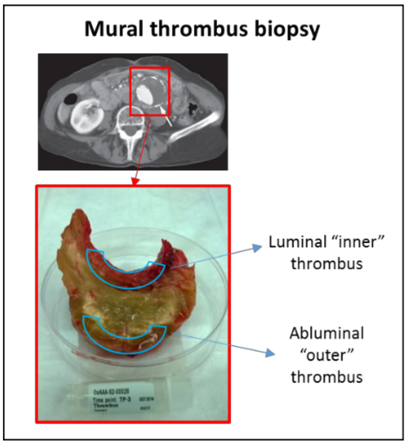

Panel B

Patient Demographics

	Patient	1	2	3	4	5	6
**ID and General Examination**	Date of Birth	29/08/33	12/01/49	10/10/33	29/05/31	12/11/45	19/04/50
	Age at Consent Date	80	65	80	83	68	65
	Sex	M	M	M	M	M	M
	Height (m)	1.64	1.70	1.67	1.69	1.78	1.88
	Weight (kg)	73.0	82.5	60.0	76.6	82.3	112.3
	BMI	27.1	28.5	21.5	26.8	26.0	31.8
	Waist (cm)	114	98	85	93	94	124
	Hips (cm)	101	97	83	100	98	109
	Waist/Hip ratio	1.13	1.01	1.02	0.93	0.96	1.14
	Never Smoked	0	0	0	0	0	0
	Ex-smoker	1	0	0	1	0	1
	Current smoker	0	1	1	0	1	0
**Cardiovascular History**	Type of tobacco consumed	Cigarettes	Cigars	Cigarettes/ Cigars	Cigarettes/ Cigars	Cigarettes	Cigarettes
	Number of years of smoking	60	30	50	15	40	10
	Pack Years	60	30	46	11.25	40	5
	For ex-smoker: stopped	5			30		40

duration (years)						
Diabetes	0	0	0	0	0	0
Diagnosis - status unknown	0	0	0	0	0	1
Diabetes - lifestyle management	0	0	0	0	0	0
Diabetes - oral medication	0	0	0	0	0	0
Diabetes - insulin medication	0	0	0	0	0	0
Hypertension	1	0	1	1	0	1
Hypercholesterolaemia	0	0	1	1	0	1
Peripheral vascular disease	0	0	0	1	0	0
History of percutaneous arterial intervention	0	0	0	0	0	0
History of peripheral arterial bypass	0	0	0	0	0	0
History of venous disease treatment	0	0	0	1	0	0
Coronary heart disease	1	0	0	1	1	1
History of stable angina	0	0	0	0	1	1
History of myocardial infarction/acute coronary syndrome	1	0	0	1	0	1
History of percutaneous coronary intervention	0	0	0	0	1	1
History of coronary artery bypass graft	0	0	0	1	0	1
Cerebral vascular disease	0	0	1	0	0	0
History of TIA, amaurosis fugax	0	0	1	0	0	0
History of stroke	0	0	0	0	0	0
Valvular heart disease	0	0	0	0	0	1
Cardiac arrhythmia	0	0	0	0	0	0
Family history of CHD	0	1	1	0	0	1
Family history of aneurisms	0	0	1	0	1	0
Alcohol consumption (units per week)	5	20	2	10	10	14

Mouse housing and genotyping

Mouse breeders were housed in isolators with 12-h light/dark cycles and controlled temperature (20 – 22 °C). Experimental mice were housed in individually ventilated cages with 12-h light/dark cycles and controlled temperature (20 – 22 °C). Access to water and standard chow was ad libitum. Fresh mouse ear tissue biopsies were lysed in mammalian cell lysis buffer (100 mM Tris pH 8.5, 5 mM EDTA, 0.2% SDS and 200 mM NaCl) containing proteinase K (3.18 µl/ml) at 52°C for one hour with agitation. Temperature was raised to 72°C for 30 min and then the digested tissue left at room temp for a further 30 min. SDS was then diluted by taking 50 µl of sample and adding 400 µl of DNAse free water. A PCR master mix was then constructed as follows: DNA sample 0.8 µl, DNAse free water 12.9 µl, green buffer mix 4 µl, MgCl2 0.8 µl, Primers 1 µl, dNTP 0.4 µl and Taq DNA polymerase 0.1 µl this gave a total volume of 20 µl. This mix was run on a PCR thermal cycler (Thermo Fisher) using the programs outlined in Supplementary Table 3.

Atherosclerosis quantification.

Mice were fed standard chow diet and killed via CO_2 inhalation at 19 weeks of age. Whole mouse aortae were perfused with 4% paraformaldehyde, dissected, trimmed of extraneous tissue, opened and pinned endothelium side up. Lipid deposition is described in Supplementary Methods. Aortae were then incubated *en face* with Sudan IV solution (Sigma-Aldrich) for 15 min. Aortic lipid deposition was assessed from digitized images of fixed aortas stained with Sudan IV dye using Image-Pro analyzer v6.3 (Media Cybernetics, USA).

Histological processing of tissue and sectioning.

Tissue samples were then transferred to 70% ethanol for storage until processing. Tissue samples were processed using a HistoCore PEARL Tissue processor (Leica) allowing for thin sectioning. The samples were processed through to paraffin wax via serial cycles of methanol which involved dehydration to remove water from the tissue allowing the infiltration of wax and xylene, to clear the sample of water and methanol. Samples then underwent permeation with wax. Paraffin embedded tissue samples were made

into wax blocks using a HistoCore Arcadia H, heated paraffin embedding station (Leica), tissue processor and histocentre. Tissue sections (7µm) were sectioned using a microtome and mounted onto SuperFrost Plus slides (ThermoFisher Scientific, UK) (3 sections per slide). Mounted slides were then placed vertically in a rack, and heated for 1 hr at 60°C to remove excess wax and then stored in a slide box until staining.

1. Masson trichrome: Visualization of muscle fibres, collagenous fibres, fibrin and erythrocytes was undertaken using Masson trichrome stain. Paraffin embedded sections were re-hydrated in ethanol (100%, 96%, 70%). Sections were treated with Haematoxylin Azophloxin solution, Tungstophosphoric acid orange G solution and Light Green SF staining. 1% acetic acid rinses were applied between each stain (Merck, Germany). Following staining, sections were dehydrated in ethanol (70%, 96%, 100%) before observation under normal light microscopy (GX Optical, MZS Series stereo microscope, GXM MZS0745TL-R).

2. Galectin-3 Macrophages were visualized using Galectin-3 in the aortic root. Paraffin embedded sections were re-hydrated in ethanol (100%, 96%, 70%, 30%) and incubated with 50µl serum free protein block (Dako, X0909) for 1 hour. Goat polyclonal anti-mouse Galectin-3 antibody (primary antibody) at 1 µg/ml PBS was added and sections incubated for 2 hours (RnD Systems, AF1197). Vector biotinylated rabbit anti-goat (secondary antibody) (Vector Laboratories, BA-5000) was added before sections were treated with ABC-Alkaline Phosphatase solution prepared according to manufacturer's instructions (Vector Laboratories, AK-5000) Vector Red Alkaline Phosphatase Substrate was prepared (in 100 mM Tris-HCl pH 8.2) according to manufacturer's instructions. Following application sections were checked for positive staining for up to 40 minutes. Murine liver was used as a positive control.

3. Alpha smooth muscle actin (α-SMA) staining. Mouse monoclonal SMα-Actin clone 1A4 (Sigma Aldrich, A5691) was used for visualization of alpha(α)-smooth muscle actin isotype in the aortic root. Paraffin embedded sections were re-hydrated appropriately in ethanol (100%, 96%, 70%, 30%) and incubated for 1 hour with 50µl serum free protein block (Dako, X0909). Sections were incubated with 50 µl SMα-Actin antibody conjugated to alkaline phosphatase (1:50 PBS dilution)

for 2 hours, then washed with PBS and Tris- HCl. Vector red alkaline phosphatase substrate was prepared (in 100 mM Tris- HCl pH 8.2) according to manufacturer's instructions (Vector Laboratories, SK-5100) and added to the sections. Normal light microscopy was utilized to monitor color development (positive staining was seen as bright red). Sections were frequently checked for up to 40 minutes at which point the reaction was terminated and the appropriate dehydration steps applied (70%, 96%, 100%). Lung tissue was used as a positive control. Positive staining is observed as intense staining (red color) around the larger airways. Murine isotype control (Abcam Mouse IgG-Isotype control, ab37355) was used to differentiate between background staining which might have occurred. Following staining, Neo-mount and coverslips were added to all sections to preserve fluorescence.

Individual aortic valves sections, taken at approximately 100-μm intervals, were imaged to calculate average lesion area for each valve using a Canon PowerShot SX60 HS 16.1 MP under x4 magnification light microscopy. Slides utilized started from the section showing all three aortic valve leaflets. Plaque morphology (Masson Trichrome staining), alpha-smooth muscle actin isotype (α-SMA), and macrophage infiltration of the valve wall (Galectin-3) were quantified using Image Pro® Analyzer 6.3.

Ang II infusion, AAA development and blood pressure recordings.

After mice were euthanized by CO_2 asphyxiation at day 14, the whole aorta was dissected from the heart to the femoral bifurcation removing any surrounding tissue. The abdominal aorta with or without AAA was classified according to Daugherty's classification(2). Two independent investigators scored aortae. AAA experiments were conducted over a long period, thus some datasets were re-used for later comparisons with additional controls undertaken at that time to confirm the model was behaving as expected. All images are provided in Supplementary Figures. We ensured that controls were processed throughout the whole period, to confirm the model was behaving as expected at all times. The aortae were then stored in 4 % PFA overnight at 4 ºC and then transferred to 70 % Ethanol for long term storage at 4ºC. Systolic blood pressure was measured every morning using a non-invasive computerized tail-cuff system in conscious

mice following a 2-week training period (Visitech BP2000, Visitech Systems Inc., USA). The mice were positioned in a magnetized restrainer which incorporated a heat pad set at 37°C.

RNA extraction, cDNA synthesis and Real Time PCR (RT-PCR)

AAA tissue (weight range 50-150 mg) were dissected from mice (n = 4 mice per group) and were weighed and then snap frozen in liquid N_2 before being stored at -80 ^{0}C. Samples were placed in cold-safe plastic tubes containing ceramic beads, 1 ml of ice cold Tri reagent (Sigma-Aldrich, UK) was added, tubes were then placed in a Bead Raptor Elite (Omni International, GA, united states) and tissue homogenised, at 7.10 m/s^2, for 2 runs of 15 sec with a 15 sec dwell time between runs, with the Raptor cooling unit at full power. 200 µl of bromochloropropane was added and sample allowed to sit on ice for 10 min, before being centrifuged at 15,000 g for 15 min at 4 ^{0}C. The upper aqueous phase was transferred to a new tube and 250 µl of 3 M sodium acetate (pH 5.5), 700 µl propanol and 10 µl glycogen added to the sample, before incubation at -20 ^{0}C overnight. Samples were next spun at 15,000 g for 15 min at 4^{0}C to pellet the RNA, the pellet was washed 3 X in 70 % ethanol, then allowed to air dry for 5 min, then resuspended in 50 µl RNAse free water. RNA samples underwent clean up to remove protein and solvent contamination using the RNeasy MinElute Cleanup Kit (Catalogue number 74204 Qiagen, MD, USA), as per manufacturers instructions. 1 µl of each sample was read on a NanoDrop™ 2000/2000c Spectrophotometers (ThermoFisher Scientific, Newport UK), to calculate RNA concentration and ensure samples were free of contamination. All RNA samples had A260/A230 values of between 1.8 and 2.2, and A260/A280 values between 1.8 to 2.1. Five RNA samples (5 µl, chosen at random), were sent to Wales Gene Park to conduct RNA integrity analysis, and all had RIN values of 7.5-9.2. For cDNA synthesis, 1 µg total RNA was reverse transcribed using RT2 First Strand Kit (catalogue number 330404, Qiagen, MD, USA), as per manufacturers instructions, to yield 112 µl of cDNA. For RT-PCR, RT^2 Profiler™ PCR Array for Mouse Inflammatory Response & Autoimmunity (catalogue number PAMM-077ZA-24, Qiagen, MD, USA) was used according to the manufacturers instructions, In brief, 102 µl of cDNA was added to 1.2 ml RNase-free water and this was combined with 1.35 ml 2x RT^2 SYBR green mastermix. The solution was vortexed and 25 µl added to plate wells containing predesigned primers for targets of interest (and five housekeeping

genes). The plate was analyzed on a Applied Biosystems™ QuantStudio™ 12K Flex Real-Time PCR system with the following settings, the plate was heated to 95 ^{0}C for 10 min, then 40 cycles were conducted (95 ^{0}C for 15 sec, then 60 ^{0}C for 1 min). In addition, a melt curve was run for each to ensure only a single peak was observed. Data was inspected to ensure that in each plate, the positive control, reverse transcription control and negative control (mouse genomic control) were within the range of the manufacturers recommendations, then statistical analysis (t-tests) conducted to ensure that no significant difference between the 5 housekeeping/reference genes (Actb, B2m, Gapdh, Gusb and Hsp90ab1), were seen. The genes of interest are analysed as the $\Delta\Delta C_T$

Phospholipid liposome preparation.

Liposomes were made by extrusion in phosphate buffered saline (PBS), pH 7.4. Control liposomes (aPL) contained 25.67 µg DSPC, 11.52 µg SAPE, 2.08 µg SAPS and for eoxPL liposomes: 3.92 µg 12-HETE-PE was added with a reduced amount of SAPE (7.68 µg). The lipids were mixed and dried in a glass vial by evaporation under $N_{2,}$ then suspended in 500 µl PBS. Total lipid concentration in 500 µl PBS was 100 mM. Human recombinant tissue factor (4.25 ng) (Cambridge Biosciences, RTF-0300-10) was added. Liposomes were then generated by 10 freeze thaw cycles with liquid nitrogen, followed by passing through LiposofastTM mini-extruder with 100 nm pore membranes (Avestin) X19 cycles. Liposomes were diluted x 40 in PBS and the final dose of 12-HETE-PE administered for tail bleeding (10 µl injection) and i.v. experiments (50 µl injection) was 10 ng with an overall lipid concentration of 2.5 µM and 106 pg human recombinant tissue factor.

Blood lipid analysis and clotting parameters.

1. Plasma thrombin/antithrombin (TAT) complexes were assayed using a murine TAT ELISA Kit, as per manufacturer's instructions (Abcam, ab137994). Whole blood was collected via cardiac puncture into $^1/_{10}$ volume of 3.8 % trisodium citrate as an anticoagulant and centrifuged at 3000 x g for 10 min. Plasma was stored at -80 °C. Plasma was diluted 1:100 and then added to each well containing TAT complexes specific antibody. TAT complexes specific biotinylated detection

antibody is added followed by Streptavidin-Peroxidase conjugate. The resultant yellow color was measuring using absorbance 450 nm. TATs were determined in male 10 – 11-week old mice from all genetically modified strains.

2. Prothrombin Time (PT) was measured using an automatic coagulometer (Amelung KC 10). Whole blood was collected from all mice outlined above into 3.8% trisodium citrate, blood was then spun at 2 x 1000g for 10 min to collect plasma and stored at -80 °C. Citrated plasma was warmed in a water bath at 37 °C for 5 min. Plasma was then incubated with 37 °C prewarmed RecombiPlasTin 2G reagent (Werfen) containing tissue factor relipidated in a phospholipid blend and calcium chloride. Plasma (40 µl) was added to a plastic cuvette along with a magnet aligned with a magnetic detector, which holds the magnet in place. The magnet remained locked in position within the cuvette while the test tube rotated. Addition of RecombiPlasTin 2G (100 µl) promoted clot formation. Once formed it entangled the magnet, breaking the electromagnetic coupling and allowing the magnet to rotate within the tube, thus terminating the test. Time for termination is designated PT.

Isolation and activation of mouse platelets

Mouse platelets were isolated as described(3). Whole blood was obtained by cardiac puncture directly into a syringe containing 150 µl of ACD [2.5% (w/v) trisodium citrate, 1.5% (w/v) citric acid, and 100 mM glucose]. The syringe was emptied into an Eppendorf tube containing 150 µl of 3.8% (w/v) sodium citrate, and 300 µl of modified Tyrode's buffer was then added (145 mM NaCl, 12 mM $NaHCO_3$, 2.95 mM KCl, 1 mM $MgCl_2$, 10 mM HEPES, and 5 mM glucose). The blood was spun for 4 min at 200 g at 25 °C, and platelet-rich plasma (PRP) was removed. Another 400 µl of Tyrode's buffer was added and carefully mixed into the blood without inverting the tube, and more PRP was removed after a second spin of 2 min at 200g. A third spin at 500g for 5 min on the pooled PRP pelleted the platelets, plasma was removed, and the platelets were resuspended in Tyrode's buffer at 2 x 10^8/ml. Half of the platelets were used as unstimulated controls, and the rest were activated with thrombin (0.2 U/ml) and 1 mM $CaCl_2$ for 30 min at 37°C.

Isolation and activation of mouse eosinophils.

Eosinophils were generated from bone marrow isolated from 8-week-old mice as previously described (4) with minor modifications. Bone marrow was incubated in RPMI (Gibco) containing 20% heat-inactivated feral calf serum (FCS), 25 mM HEPES, 100 IU/ml Penicillin (Gibco), 10 µg/ml Streptomycin (Gibco), 2 mM glutamine (Gibco), 1x NEAA (Sigma), 1mM Sodium pyruvate (Sigma), 50 µM β-mercaptoethanol (Gibco), 100 ng/ml mFLT3L (Peprotech) and 100 ng/ml mSCF (Peprotech) for 4 days, followed by 10 days differentiation with 10 ng/ml IL-5 (Peprotech). Half of the medium was changed every other day. Maturation was monitored by flow-cytometry of SiglecF and CCR3 expression. Fully matured eosinophils were used after 14 days of total culture. For thrombin generation assays, RAW 264.7 mouse macrophages were treated with 100 ng/ml LPS in RPMI containing 10% FCS for 24 hrs.

Externalization of PE or PS on the surface of platelets and eosinophils

Biotinylated standards (DMPE-B, DMPS-B) were generated as described previously(5). For biotinylation of total PE and PS, 100 µl platelet sample ($2x10^6$ platelets) was added to 20 µl of 6.82 mg/ml EZ-link NHS-Biotin (Thermo-Fisher Scientific, UK) dissolved in dimethyl sulfoxide (Sigma-Aldrich, UK). For external PE/PS, 100 µl platelet sample ($2x10^6$ platelets) was added to 86 µl EZ-link sulfo-NHS-Biotin dissolved PBS at a concentration of 5 mg/ml. Both samples were then incubated for 10 min at room temperature. Lysine was dissolved in PBS (36.6 mg/ml) and 72 µl was then added to the platelet suspension containing EZ-link sulfo-NHS-Biotin to quench the reaction for a further 10 min. Internal standards were added: 10 ng DMPS-B, DMPE-B, DMPC and DMPE per sample. PLs were extracted using the method described by Bligh and Dyer(6). For each 400 µl sample 1.5 ml of 1:2 (v/v) $CHCl_3$:MeOH was added and the sample vortexed. Then, 0.5 ml of $CHCl_3$ was added and again vortexed. Finally, 0.5 ml of HPLC-grade H_2O was added, vortexed and centrifuged at 500*g* for 5 min at room temperature. The lower phase was recovered using a glass Pasteur pipette then evaporated to dryness using a Rapidvap N2/48 evaporation system (Labconco Corporation) and re-suspended in 200 µl MeOH. Extracts were stored at -80 °C until LC/MS/MS analysis. Mass spectrometry for biotinylated PE and PS was performed on a Q Trap 4000 (AB Sciex UK Limited, Warrington, Cheshire, UK) as described previously(5). Phospholipids were separated

by reverse-phase HPLC-electrospray ionization (ESI)-MS of biotinylated APL using an Acentis C18 column (5 µm, 150 x 2.1 mm, Sigma-Aldrich) with the following conditions: temperature, 22 °C; flow rate, 400 µl min^{-1}; isocratic mobile phase, MeOH with 0.2 % (wt/vol) ammonium acetate for 25 min.

Externalization of PE and PS species in eosinophils was measured according as previously described(5). In brief, cultured mouse eosinophils (4×10^6 per ml) were stimulated with ADP (40 µM) and treated with EZ-link NHS-biotin or EZ-link sulfo-NHS-biotin (Thermo Fisher Scientific) for measuring total cellular lipids and external aminophospholipids, respectively, by LC/MS/MS, with extraction and analysis as described for platelets above.

Clot formation using mouse blood

To model physiological clot formation, whole mouse blood was anticoagulated using citrate and corn trypsin inhibitor to prevent the contact pathway. Coagulation was initiated by re-calcification at 37 °C for up to 3 hrs, and the use of glassware avoided at all times, before the clot was harvested for lipid extraction and analysis. For this, blood was collected with 3.8 % sodium citrate (9:1, v/v) and 0.1 mg/ml CTI via cardiac puncture, after CO_2 euthanasia, from mice at 15 – 19 weeks of age. 200 µl of whole blood was then incubated at 37 °C in the absence or presence of 20 mM $CaCl_2$, 2 µM liposomes (5 % SAPS, 65 % DSPC and 30 % SAPE) containing 5 pM recombinant tissue factor (rTF), for up to 3 hours. Samples were then spun at 16,000 x g for 5 min at 4 °C, serum removed, cell pellets immediately snap frozen in liquid nitrogen and stored at − 80 °C before lipid extraction. Unstimulated blood aliquots were immediately spun, plasma removed, cells snap frozen and analyzed for eoxPL basal levels. Extraction and analysis of lipids from clots is described in full in Supplementary Methods.

Tissue processing for mouse and human AAA tissue.

Tissue (human AAA or thrombus, or murine AAA) was weighed then transferred to an Eppendorf tube containing 0.5 ml buffer (phosphate buffered saline, pH 7.4 containing 100 µM diethylenetriamine pentaacetate (chelator) and butylated hydroxytoluene (antioxidant), with 7.5 µM acetaminophen (prevent

Hb redox cycling) also added to human samples). 10 ceramic beads were added with 1 mM $SnCl_2$, then the sample homogenized in a Bead Ruptor Elite at 4 °C using 5 rounds of homogenization: 3 x(4.5 m/s, 1 cycle, 15 s) and 2 x(3.5 m/s, 1 cycle, 15 s). Macerated tissue and beads were removed into a glass extraction vial, and the Eppendorf rinsed with 0.5 ml buffer, which was then added to the sample. Lipids were extracted using a double extraction method as follows: First, lipids were extracted by adding a solvent mixture (1 M acetic acid, 2-propanol, hexane (2:20:30)) to the sample at a ratio of 2.5 ml solvent mixture to 1 ml sample, vortexing and then adding 2.5 ml of hexane. Following vortexing and centrifugation (400 g, 5 mins), lipids were recovered in the upper hexane layer. The samples were then re-extracted by the addition of an equal volume of hexane followed by further vortexing and centrifugation. Vortex each sample for 1 min. The combined hexane layers were then dried under vacuum. Next, 2.5 ml methanol and 1.25 ml chloroform was added, and sample vortexted for 1 min. 1.25 ml chloroform and 1.25 ml water was then added. After vortexing and centrifugation (400 g, 5 mins), lipids were recovered in the bottom chloroform layer. The chloroform layer was dried, dissolved in methanol, and stored at − 80 °C before analysis by LC/MS/MS.

Lipid extraction and analysis of clots

100 mg clots were homogenized in 1 ml ice cold PBS, pH 7.4, containing 100 μM diethylenetriaminepentaacetic acid, 100 μM butylated hydroxytoluene and 7.5 μM acetaminophen using a disposable double-ended polypropylene micro pestle. Hydroperoxides were reduced to their corresponding stable alcohols by the addition of 1 mM $SnCl_2$ for 10 min on ice. 1,2-dimyristoyl-PE and -PC (5 ng) were added to each sample before extraction as internal standards. Lipids were extracted and analyzed using LC/MS/MS as described below. HETE-PEs were quantified as outlined previously (1). 100 mg clots were homogenized in 1 ml ice cold PBS, pH 7.4, containing 100 μM diethylenetriaminepentaacetic acid, 100 μM butylated hydroxytoluene and 7.5 μM acetaminophen using a disposable double-ended polypropylene micro pestle. Hydroperoxides were reduced to their corresponding stable alcohols by the addition of 1 mM $SnCl_2$ for 10 min on ice. 1,2-dimyristoyl-PE and -PC (5 ng) was added to each sample before extraction as internal standards. Lipids were extracted by adding 2.5 ml of methanol and 1.25 ml of chloroform to 1 ml

of sample, followed by 1 min vortex and incubation on ice for 15 min. Then, 1.25 ml of chloroform and 1.25 ml of water was added. After vortexing and centrifugation (400 g, 5 mins), lipids were recovered in the bottom chloroform layer. The chloroform layer was dried, dissolved in methanol, and stored at − 80 °C before analysis by LC/MS/MS.

LC/MS/MS analysis of eoxPL

Lipid extracts were separated using reverse-phase HPLC on a Luna 3 μm C18 150 × 2-mm column (Phenomenex, Torrance, CA) with a gradient of 50 – 100 % B over 10 min followed by 30 min at 100 % B (A, methanol:acetonitrile:water, 1 mM ammonium acetate, 60:20:20; B, methanol, 1 mM ammonium acetate) with a flow rate of 200 μl min^{-1}. Products were analyzed in MRM mode, on a 6500 Q-Trap (Sciex, Cheshire, United Kingdom), monitoring transitions from the parent ion to daughter ion (dwell 75 ms) with TEM 500 °C, GS1 40, GS2 30, CUR 35, IS − 4500 V, DP − 50 V, EP − 10 V, CE − 38 V and CXP at − 11 V. The area under the curve for the parent ion to daughter ion was integrated and normalized to the internal standard for lipid species described in Supplementary Table 4. For quantification of 5-, 8-, 11-, 12- and 15-HETE-PEs, standard curves were generated with purified PE(18:0a/5-HETE), PE(18:0a/8-HETE), PE(18:0a/11-HETE), PE(18:0a/12-HETE) and PE(18:0a/15-HETE) synthesized as described previously(1). All eoxPL shown herein were recently identified to form in human blood during clotting, with all structures confirmed using MS/MS, and assignment of fragment ions from the PL and the *Sn2* oxidized fatty acid. Where specific internal daughter ions were detected, the site of oxygen insertion is named, otherwise, the number of carbons, rings/double bonds and oxygens attached at the *Sn2* fatty acid is provided(7). Where more than one eoxPL with the same structure is listed, this denotes additional molecular species with different retention times.

Tail bleeding assay

All mice were kept in constant temperature cages (20 – 22 °C) and given free access to water and standard chow. Mice were anesthetized using 5% isoflurane and maintained with 2% isoflurane. Where administered, 50 μl liposomes made as described above were injected intravenously in (10 ng 12-HETE-

PE/injection). After 1 hr, the tail was transected 5 mm from the distal end and immediately immersed in 37 °C physiological saline. Bleeding was observed as blood loss and time for stable cessation of blood flow, before killing via cervical dislocation. Hemoglobin (Hb) content of the saline was determined as follows: Hemoglobin quantitation was achieved via centrifugation of the tube at 250 x g for 15 min, and re-suspending red cells in 5 ml erythrocyte lysis buffer (8.3 g/L NH_4Cl, 1 g/L $KHCO_3$ and 0.037 g/L EDTA in distilled H_2O). The concentration of Hb was measured as optical density (OD) 575 nm using a UVIKON 923 double beam UV /VIS spectrophotometer (Bio-Tek Kontron Instruments) and expressed as absorbance units (AU).

Heatmap and Cytoscape correlation

For generation of heatmaps, first the samples with $n \geq 3$ were averaged. Next, log10 was applied to the analyte to internal standard ratio normalized to tissue weight (mg) for each eoxPL. R's Pheatmap package was used to generate the heatmap plots. The Euclidean metric was chosen to establish the treatments' relationships depicted as clusters(8). The clusters are aggregated following the "shortest distance" rule. Lipids are color-coded by PL headgroup. Relationships between related lipids were visualized in Cytoscape (version 3.6.0) using Pearson correlations generated with R ($r > 0.8$).

Statistical analysis.

Between-group comparisons of normally distributed measurements were assessed by Student's t-test. The Mann Whitney-U test was applied to examine differences between non-parametrically distributed variables. One-way analysis of variance was used to compare more than two data groups. The Fisher's exact test was used to examine the significance of association between two kinds of classification (e.g. AAA incidence). For the Cytoscape network, lipids were identified as *Alox12*–derived based on their complete absence in blood clots from *Alox12*$^{-/-}$ and *ApoE*$^{-/-}$/*Alox12*$^{-/-}$ mice and assessed using the Kruskal–Wallis test for non-parametric distributions (cut-off adjusted $P < 0.05$, Benjaminin-Hochberg correction). Next, time-dependent changes in total HETE-PE levels in blood clots from *ApoE*$^{-/-}$, *Alox12*$^{-/-}$ and *Alox15*$^{-/-}$ were

compared to wild type mice, and *ApoE*$^{-/-}$/*Alox12*$^{-/-}$ and *ApoE*$^{-/-}$/*Alox15*$^{-/-}$ were compared to *ApoE*$^{-/-}$ mice by log-linear regression model with mouse strain as a categorical variable and time as a continuous variable. Each time point (0 – 180 min) measurement for eoxPLs represents n = 3, mean ± SEM. $P < 0.05$ was considered statistically significant. For quantification of total HETE-PEs, 5-, 8-, 11-, 12- and 15-HETE-PEs were individually quantified and then summed for each time point.

- *Study Design.* Sample size was determined based on previous experience with similar mouse experiments, and power calculations undertaken by the Channon group. Numbers were not generally altered during studies.
- *Rules for stopping data collection*. No rules were defined for stopping data collection
- *Data inclusion/exclusion criteria*. No criteria were applied, all data generated has been included.
- *Outliers*. No outliers were excluded
- *Selection of endpoints*. End points were defined based on observation of AAA development, using a standard 2-week model.
- *Replicates*. Information on number of replicates (biological and technical) has been provided in all experiments in Figure legends. As these experiments are performed on animals, each n represents a different animal for most experiments. For ethical reasons, we do not repeat more than necessary to ensure that the lowest number of mice are used in our experiments.
- *Research objectives*. Our hypothesis was that eoxPL play a role in vascular inflammatory diseases, specifically atherosclerosis and AAA. When we discovered the phenotype, we then focused on the idea that this was driven by coagulation because we had observed potent hemostatic activities of the lipids in ongoing studies.
- *Research subjects or units of investigation*. Mice (genetically-altered strains) and cells/blood isolated from these mice.
- *Experimental design.* Controlled laboratory experiments in vivo and in vitro. Treatments applied included using genetically-altered strains and administration of drugs and lipids by osmotic minipump.

Observations included development of atherosclerosis, AAA, changes in hemostatic parameters and bleeding.

- *Randomization*. Mice were assigned to units based on their genotype. Data was not processed randomly, but with all experimental groups together.
- *Blinding*. Some aspects of the study were blinded, specifically the analysis of the immunohistochemistry data, the aneurysm classification and the TAT ELISAs.

Supplementary Results

Alox12 or Alox15 deficiency in ApoE$^{-/-}$ mice reduces atherosclerosis

AAA form in vessels affected by atherosclerosis, therefore, prior to inducing AAA formation, we measured the development of atherosclerosis in *Alox* deficient strains when backcrossed to an ApoE$^{-/-}$ background. Plaque was quantified in the aorta and aortic root, in normal chow fed 19-week old male and female *ApoE*$^{-/-}$, *ApoE*$^{-/-}$/*Alox12*$^{-/-}$ and *ApoE*$^{-/-}$/*Alox15*$^{-/-}$ mice. En face analysis confirmed that *Alox15* deletion reduces total aortic plaque area (Supplementary Figure 2 A) (9-12). Deletion of *Alox12* similarly decreased burden in male and female mice (Supplementary Figure 2 A). Lesion size (Masson trichrome), macrophage content (Galectin-3) and smooth muscle alpha actin (SMα-actin) in the aortic root were unaffected by deletion of either isoform, indicating protection by *Alox* deletion was primarily in the whole aorta (Supplementary Figure 2 B). There was no impact of *Alox* deletion on total cholesterol in *ApoE*$^{-/-}$ mice (Supplementary Table 1).

	Total Cholesterol (mmol/l)	HDL (mmol/l)	LDL (mmol/l)	Triglycerides (mmol/l)
ApoE$^{-/-}$ male	16.63 ± 0.44	1.26 ± 0.10	7.51 ± 0.23	2.46 ± 0.29
ApoE$^{-/-}$ female	15.48 ± 0.86	1.25 ± 0.05	8.97 ± 0.29	1.71 ± 0.19
ApoE$^{-/-}$/Alox15$^{-/-}$ male	17.30 ± 1.85	1.20 ± 0.08	8.64 ± 0.31	2.20 ± 0.29
ApoE$^{-/-}$/Alox15$^{-/-}$ female	17.12 ± 0.96	1.48 ± 0.05	7.16 ± 0.29	2.43 ± 0.08
ApoE$^{-/-}$/Alox12$^{-/-}$ male	18.59 ± 1.58	1.48 ± 0.10	7.35 ± 0.31	2.82 ± 0.36
ApoE$^{-/-}$/Alox12$^{-/-}$ female	18.39 ± 1.49	1.19 ± 0.04	9.11 ± 0.38	1.99 ± 0.36

Supplementary Table 1. Total cholesterol, HDL, LDL and total triglycerides were determined as described in Methods. Data are expressed as mean ± SEM. Total cholesterol, HDL, LDL and triglyceride levels were not significantly different between genotypes. Data was analysed using a Mann-Whitney non parametric U test.

	Total Cholesterol (mmol/l)	HDL (mmol/l)	LDL (mmol/l)	Triglycerides (mmol/l)
$ApoE^{-/-}$ male Ang II	19.39 ± 1.19	2.19 ± 0.12	7.69 ± 0.31	2.52 ± 0.29
$ApoE^{-/-}$ female Ang II	16.21 ± 0.81	1.63 ± 0.11	9.02 ± 0.26	1.46 ± 0.09
$ApoE^{-/-}$/$Alox15^{-/-}$ male Ang II	18.82 ± 1.16	2.00 ± 0.09	6.98 ± 0.21	2.69 ± 0.22
$ApoE^{-/-}$/$Alox15^{-/-}$ female Ang II	13.70 ± 0.86	1.61 ± 0.06	8.04 ± 0.32	1.69 ± 0.11
$ApoE^{-/-}$/$Alox12^{-/-}$ male Ang II	19.04 ± 1.22	1.96 ± 0.08	7.06 ± 0.29	2.88 ± 0.26
$ApoE^{-/-}$/$Alox12^{-/-}$ female Ang II	13.99 ± 1.40	1.40 ± 0.14	8.23 ± 0.61	1.35 ± 0.18

Supplementary Table 2. Total cholesterol, HDL, LDL and total triglycerides were determined as described in Methods, in mice administered Ang II for 2 weeks. Data are expressed as mean ± SEM. Total cholesterol, HDL, LDL and triglyceride levels were not significantly different between genotypes. Data was analysed using a Mann-Whitney non parametric U test.

Supplementary Scheme 1. Abdominal aortic aneurysm in mice requires clotting factor activation and can be prevented by diverting coagulation from the vessel wall. *Panel A. $ApoE^{-/-}$ mice administered Ang II develop AAA, which can be prevented using pro-coagulant liposomes. Panel B. Alox-deficient mice display a consumptive coagulopathy. Panel C. Backcrossing to Alox-deficiency protects $ApoE^{-/-}$ mice from AAA development.*

Supplementary Figure 1. 12- and 15-LOX-derived eoxPL are detected in human AAA thrombus. *Representative chromatograms show detection of 16:0p/HETE-PE or 18:1p/HETE-PE molecular species, indicating a predominance of eoxPL originating from either Alox15 or Alox12.* Left panels shows chromatograms for *m/z* parent/319.2, detecting all HETE-PE positional isomers, and revealing two

prominent products in each case. These are confirmed to be 15-HETE-PE (13.7 min) or 12-HETE-PE (14.2 min), by detecting internal daughter ions at *m/z* 219.1 or 179.1 respectively.

Supplementary Figure 2. *Alox15* or *Alox12* deficiency protects against atherosclerosis development in ApoE$^{-/-}$ mice *in vivo*. *Panel A. Aortic lipid deposition is reduced by Alox12 or Alox15 deficiency in male or female mice.* *ApoE*$^{-/-}$, *ApoE*$^{-/-}$/*Alox12*$^{-/-}$ and *ApoE*$^{-/-}$/*Alox15*$^{-/-}$ 19-week old male or female mice were maintained on a normal chow diet. The aortae were then harvested and incubated *en face* with Sudan IV dye for 15 minutes, as described in Methods. *Left Panel*: representative aortae from male and female mice. *Right panel*: Atherosclerosis was quantified as percentage of Sudan IV area in whole aortae. Summary data shows individual mice. (n = 6-15) Data expressed as mean ± S.E.M, *** $p<0.001$, ** $p<0.01$, * $p<0.05$ calculated using the Student's t-test. *Panel B. Aortic root atherosclerosis is not altered by Alox12 or Alox15 deficiency.* *ApoE*$^{-/-}$, *ApoE*$^{-/-}$/*Alox12*$^{-/-}$ and *ApoE*$^{-/-}$/*Alox15*$^{-/-}$ 19-week old male or female mice were maintained on a normal chow diet. Atherosclerotic plaque deposition was quantified in the aortic valve. *Top left panel*: Phase contrast images of representative aortic lesions, stained using massons trichrome allowing visualization of the morphology of the aortic valve. *Bottom left panel*: Quantification of mean lesion size. *Top right panel*: Galectin-3 positive macrophages were quantified in the aortic valves. Microscopy depicts Galectin-3 stained aortic lesions. *Bottom right panel*: SMα-actin content in the aortic root was quantified. Each symbol represents an individual mouse (n=6–15 per group). Scale bars indicate 0.5mm for aortic roots. Data expressed as mean ± S.E.M, *** $p<0.001$, ** $p<0.01$, * $p<0.05$ calculated using a Mann-Whitney non parametric U test (NS: not significant).

Supplementary Figure 3. Photos of aortae summarized in Figure 2 B, obtained from *ApoE*$^{-/-}$ mice backcrossed with either *Alox12* or *Alox15* deficient mice, and administered Ang II for 2 weeks, as outlined in Methods.

Supplementary Figure 4. Photos of aortae summarized in Figure 2 C, obtained from *ApoE$^{-/-}$* mice backcrossed with either *Alox12* or *Alox15* deficient mice, and administered Ang II for 2 weeks, as outlined in Methods.

Supplementary Figure 5. Photos of sham controls administered PBS in place of Ang II.

Supplementary Figure 6. *Alox12* or *Alox15*-deletion does not alter Ang II-dependent blood pressure changes, while FXa activity is required for development of AAA. *Panel A. Ang II infusion led to similar elevations in blood pressure, regardless of Alox12 or Alox15 -status.* Systolic blood pressure was determined using tail cuff plethysmography during 14-day Ang II (1.1 mg kg^{-1} per day) infusion period (n = 5 – 10 per group, data presented as percentage abdominal aortic aneuryms and mean ± SEM. *Panel B. FXa activity is required for development of AAA in ApoE$^{-/-}$ mice.* 19 - 24 week old male *ApoE$^{-/-}$* mice were maintained on a normal chow diet and infused with Ang II by osmotic minipump for two weeks before Schedule 1, as described in Methods. Mice were pre- administered Rivaroxaban (5 mg.kg^{-1} per day) via chow for one day and then every day during the 2-week Ang II infusion period. *Left panel*: Representative aortae are shown. Aortae shown on the left are two additional Ang II infused mice, which were added to the full dataset shown in Figure 2B (Supplementary Figure 4 left panel) to generate the bar shown (n=17). The full dataset of Rivaroxaban-administered mice are shown above (n=6). *Right panel*: Summary data with AAA expressed as percentage incidence of the overall group (n = 6 – 22). Data from panel A was analyzed using one-way analysis of variance and Student's t-test and data from panel B was analysed using Fisher's exact test, *** p<0.001, ** p<0.01, NS = not significant.

Supplementary Figure 7. HETE-PEs increase during coagulation in *ApoE$^{-/-}$* blood, but decrease in *Alox12$^{-/-}$* and *Alox15$^{-/-}$*, while multivariate and correlation analysis of eoxPL during clot formation reveals highly regulated networks. *Panel A. HETE-PEs show elevations in ApoE$^{-/-}$ mice, which are suppressed by Alox12 or ALOX15-deficiency.* Clots were generated as in Figure 5. HETE-PEs were quantified as individual molecular species and then combined. Time-dependent changes in HETE-PE levels

in blood clots from *ApoE*$^{-/-}$, *Alox12*$^{-/-}$ and *Alox15*$^{-/-}$ were compared to wild type mice while *ApoE*$^{-/-}$/*Alox12*$^{-/-}$ and *ApoE*$^{-/-}$/*Alox15*$^{-/-}$ were compared to *ApoE*$^{-/-}$mice. Each time point represents n = 3, ± SEM. * $p \leq 0.05$, ** $p \leq 0.01$,*** $p \leq 0.001$, by log-linear regression. *Panel B. Principal Component Analysis (PCA) of eoxPL temporal generation in mouse blood clotting shows relatedness of samples at different timepoints.* 3D-PCA showing mouse strains separation by time-dependent generation of eoxPLs during *ex vivo* blood clotting ("0" indicates unstimulated/unclotted blood, "5 – 180" indicates time in minutes that blood was allowed to clot). Pink: wild type, dark blue: *ApoE*$^{-/-}$, light blue: *Alox12*$^{-/-}$, grey: *ApoE*$^{-/-}$/*Alox12*$^{-/-}$, red: *Alox15*$^{-/-}$, green: *ApoE*$^{-/-}$/*Alox15*$^{-/-}$ mouse blood. Solid arrow denotes t = 0 min, dashed arrow denotes t = 180 min. *Panel C. Corresponding loading plot shows that lipids exert influence on correlations depending on Sn2 fatty acid*. PC 1 and PC 2 are first and second principal components, respectively, with their percentage of explained variance. Green circle, oxidized phosphatidylcholine (eoxPC); Blue circle, oxidized phosphatidylethanolamine (eoxPE). *Panel D. Correlation network analysis reveals eoxPL group depending on enzymatic origin.* Lipids were identified as 12-LOX–derived based on their complete absence in blood clots from both *Alox12*$^{-/-}$ and *ApoE*$^{-/-}$/*Alox12*$^{-/-}$ mice. eoxPLs were assigned as LOX or 5-LOX as described in Materials and Methods. Cytoscape 3.6.0 correlation was performed (correlation > 0.8) using data shown in Figure 1, with nodes as individual lipids and size determined by the number of links to others. Edge thickness represents the extent of the correlation between individual nodes. eoxPLs identified as originating from *Alox12*, as determined with the Cytoscape network, were examined for statistically significant differences using the Kruskal–Wallis and Benjamini-Hochberg Post Hoc test. Green: 12-LOX, pink: 5-LOX, blue: LOX, light purple: unknown enzyme.

Supplementary Figures 8-10. Aortae from mice administered PBS. These mice were administered PBS by osmotic minipump, acting as sham controls for Ang II induced AAA generation. Some are shown in Figure 2 B,C as representative but they were not included in the full bar chart in that Figure. We show the full set of photos for all groups for completeness.

REFERENCES

1. Morgan AH, *et al.* (2010) Quantitative assays for esterified oxylipins generated by immune cells. *Nat Protoc* 5(12):1919-1931.
2. Daugherty A, Manning MW, & Cassis LA (2001) Antagonism of AT2 receptors augments angiotensin II-induced abdominal aortic aneurysms and atherosclerosis. *Br J Pharmacol* 134(4):865-870.
3. Lauder SN, *et al.* (2017) Networks of enzymatically oxidized membrane lipids support calcium-dependent coagulation factor binding to maintain hemostasis. *Sci Signal* 10(507):eaan2787.
4. Dyer KD, *et al.* (2008) Functionally competent eosinophils differentiated ex vivo in high purity from normal mouse bone marrow. *Journal of immunology* 181(6):4004-4009.
5. Thomas CP, *et al.* (2014) Identification and quantification of aminophospholipid molecular species on the surface of apoptotic and activated cells. *Nat Protoc* 9(1):51-63.
6. Bligh EG & Dyer WJ (1959) A rapid method of total lipid extraction and purification. *Canadian journal of biochemistry and physiology* 37(8):911-917.
7. Slatter DA, *et al.* (2016) Mapping the Human Platelet Lipidome Reveals Cytosolic Phospholipase A2 as a Regulator of Mitochondrial Bioenergetics during Activation. *Cell Metab* 23(5):930-944.
8. Kolde R (2012) Pheatmap: pretty heatmaps.
9. Cyrus T, *et al.* (1999) Disruption of the 12/15-lipoxygenase gene diminishes atherosclerosis in apo E-deficient mice. *J Clin Invest* 103(11):1597-1604.
10. Poeckel D, Zemski Berry KA, Murphy RC, & Funk CD (2009) Dual 12/15- and 5-lipoxygenase deficiency in macrophages alters arachidonic acid metabolism and attenuates peritonitis and atherosclerosis in ApoE knock-out mice. *The Journal of biological chemistry* 284(31):21077-21089.
11. Reilly KB, *et al.* (2004) 12/15-Lipoxygenase activity mediates inflammatory monocyte/endothelial interactions and atherosclerosis in vivo. *The Journal of biological chemistry* 279(10):9440-9450.
12. Rong S, *et al.* (2012) Macrophage 12/15 lipoxygenase expression increases plasma and hepatic lipid levels and exacerbates atherosclerosis. *Journal of lipid research* 53(4):686-695.

Supplementary scheme 1

A *ApoE$^{-/-}$* mice administered Ang II

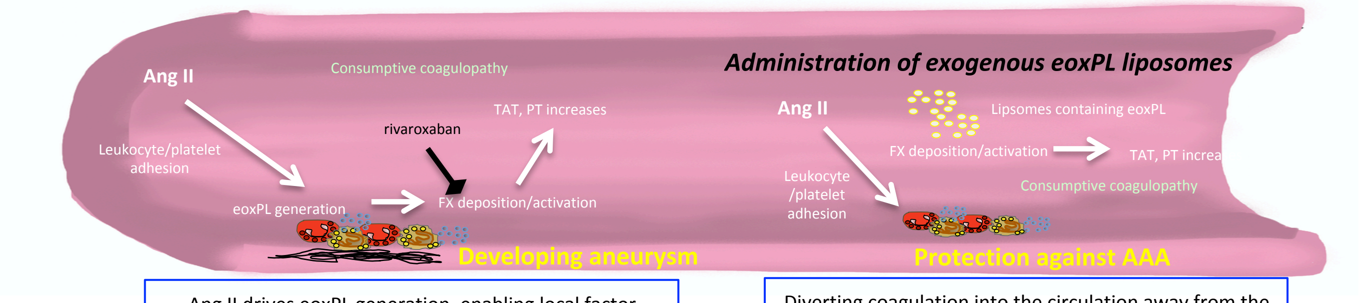

Ang II drives eoxPL generation, enabling local factor deposition/activation in *ApoE$^{-/-}$* mice

Diverting coagulation into the circulation away from the vessel wall dampens AAA development

B *Alox12$^{-/-}$ or Alox15$^{-/-}$* mice

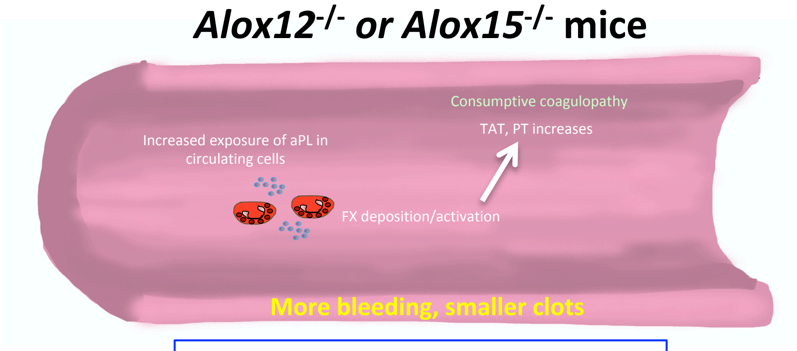

Chronic activation and removal of factors in the circulation leading to coagulopathy

C *ApoE$^{-/-}$* mice backcrossed with *Alox$^{-/-}$* mice administered Ang II

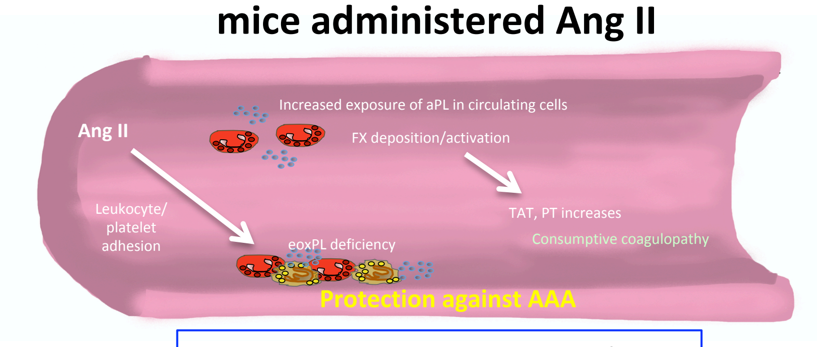

Diverting coagulation into the circulation away from the vessel wall dampens AAA development

Supplementary Figure 1

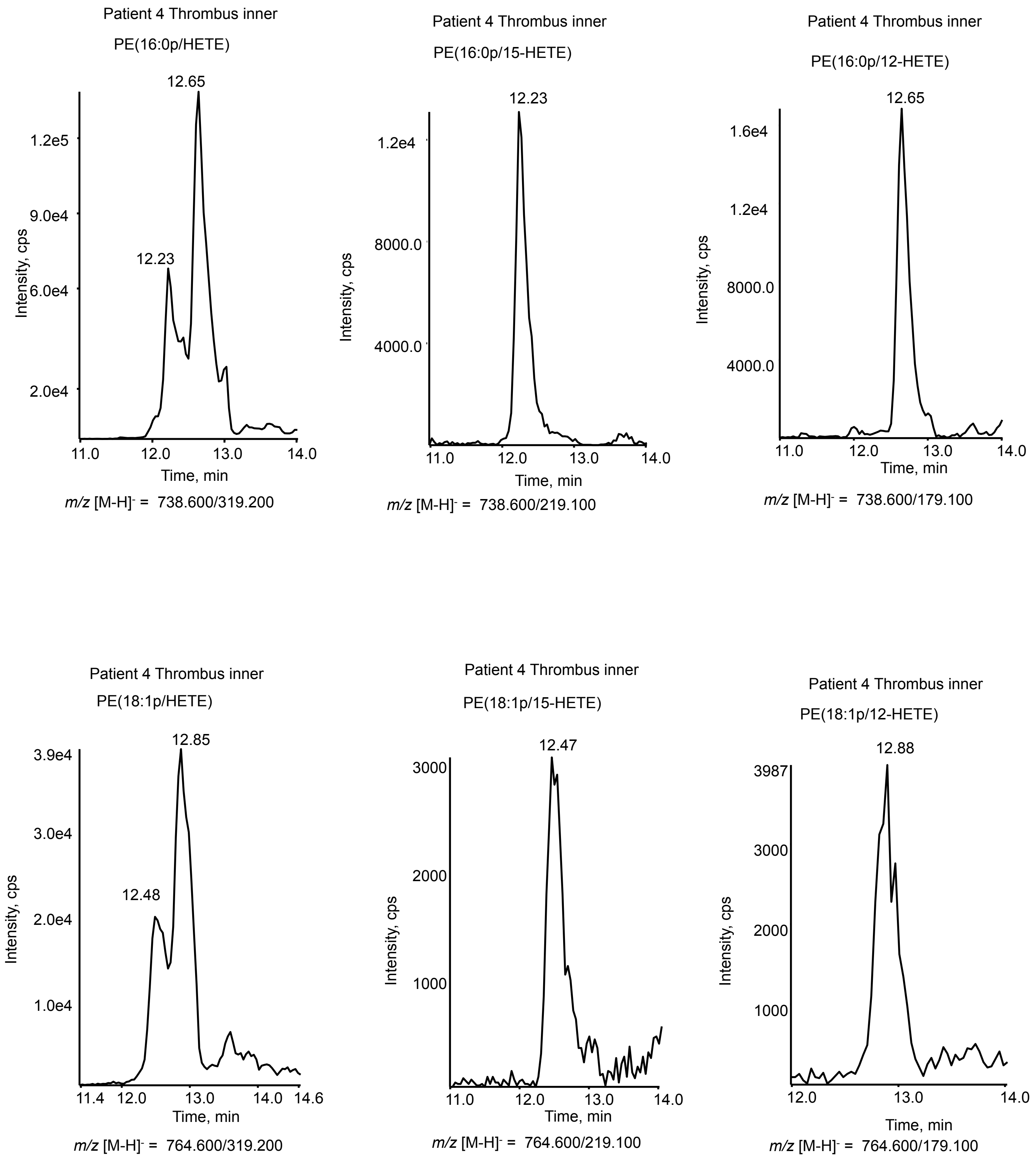

Supplementary Figure 2

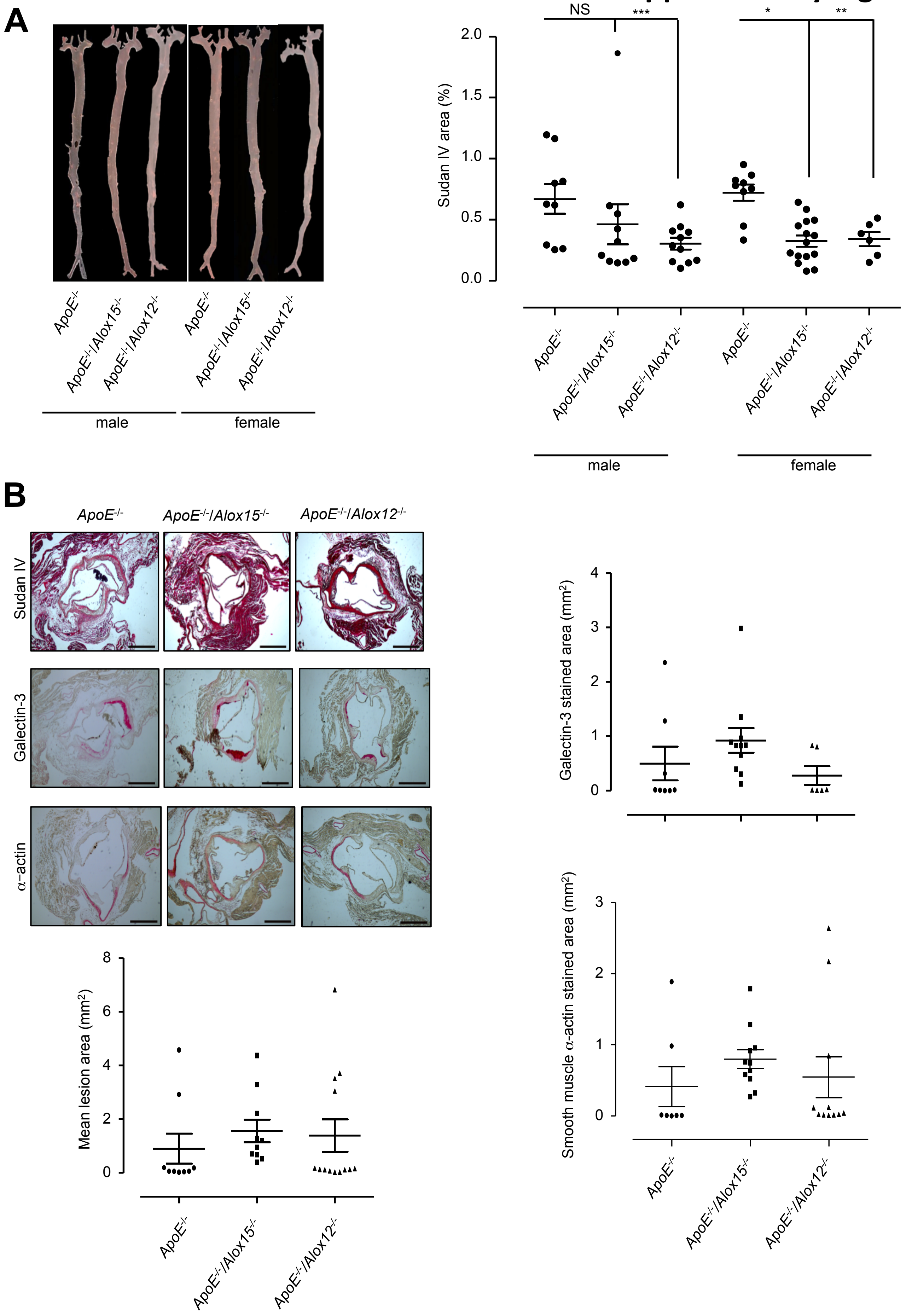

Supplementary Figure 3

ApoE$^{-/-}$ male

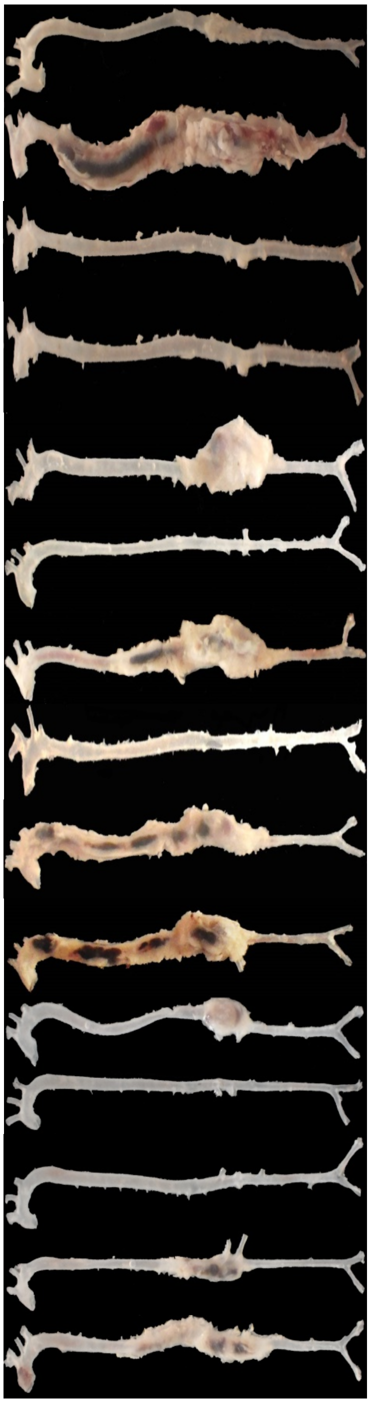

ApoE$^{-/-}$/ *Alox*15$^{-/-}$ male

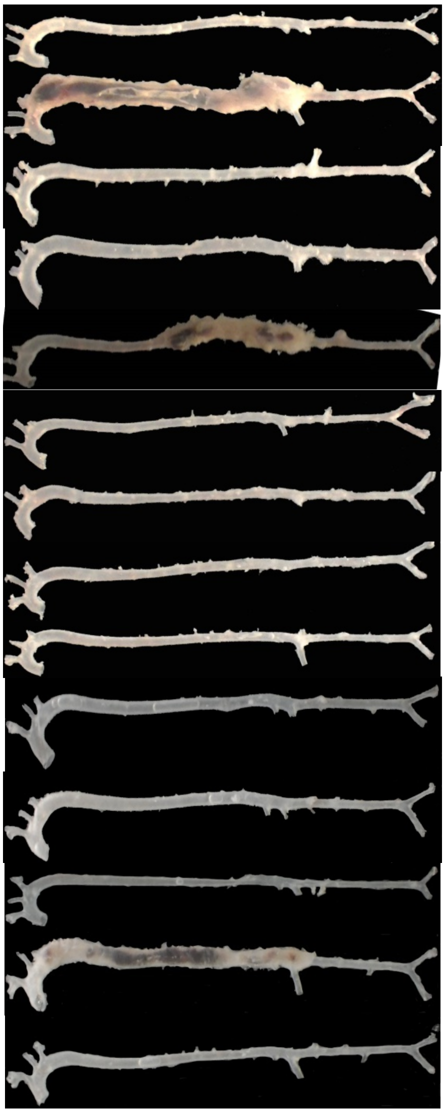

ApoE$^{-/-}$/*Alox12*$^{-/-}$ male

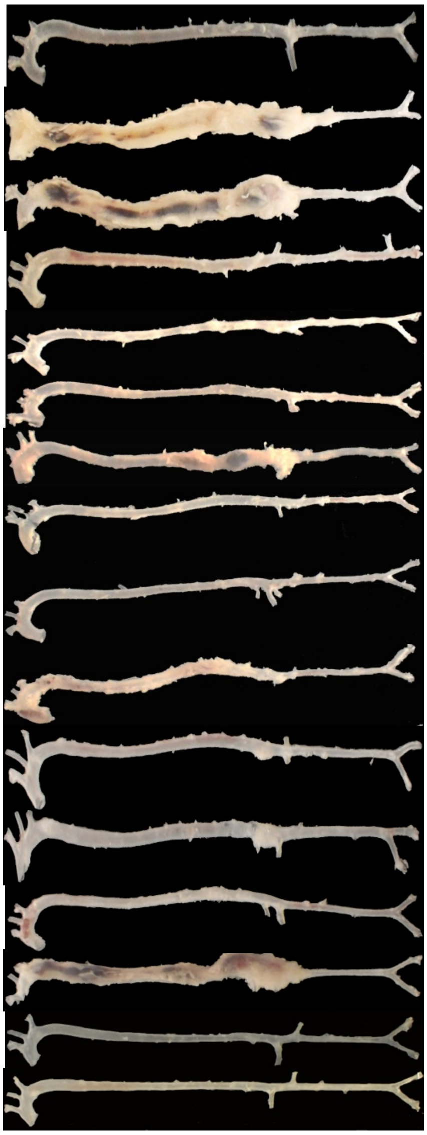

Supplementary Figure 4

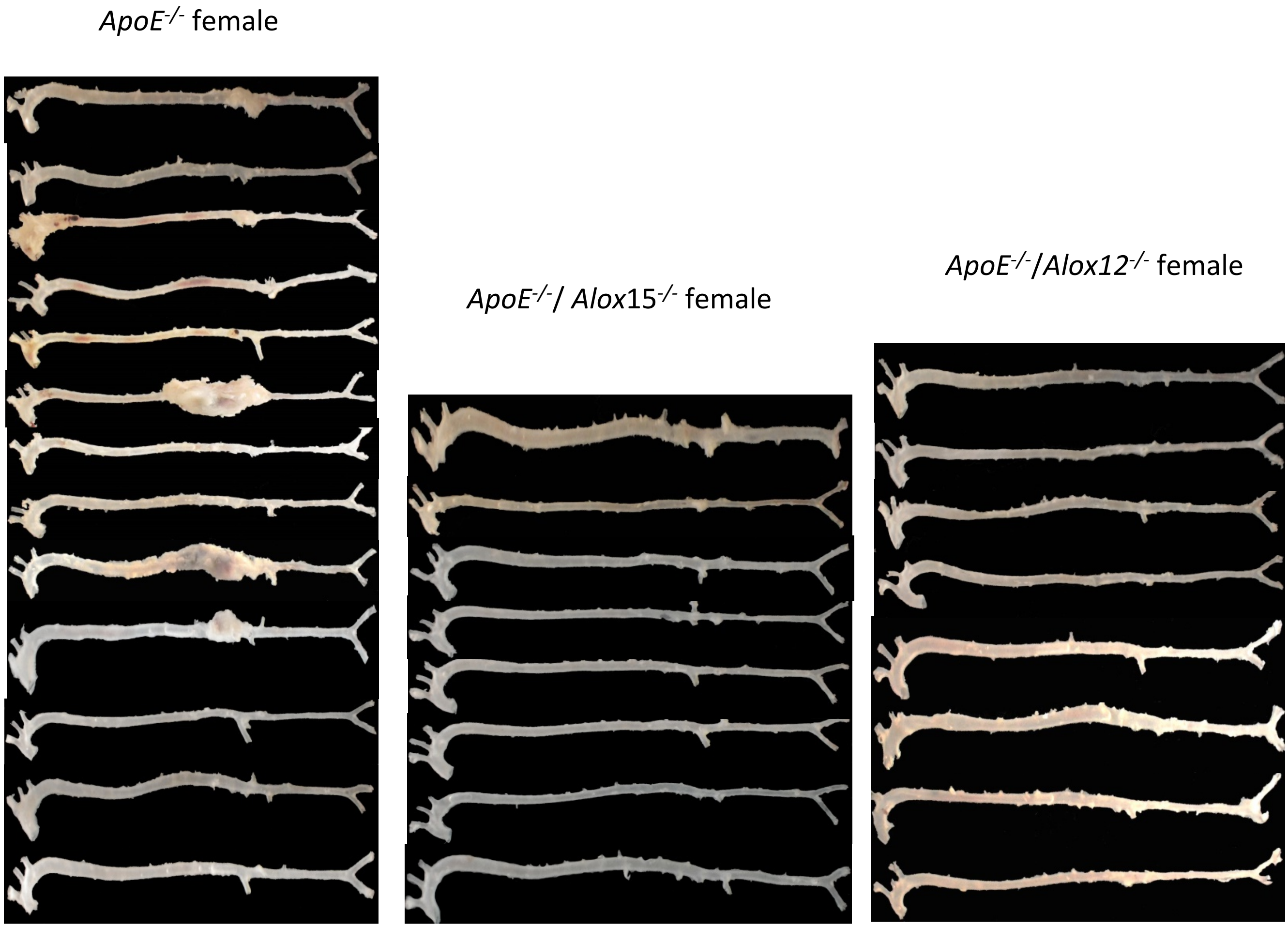

Supplementary Figure 5

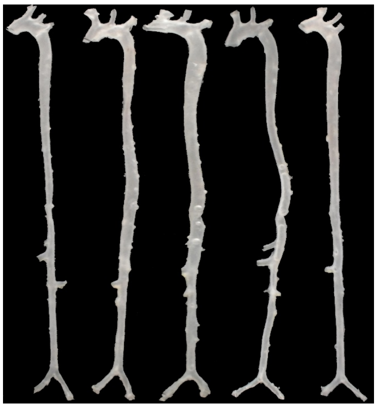

ApoE$^{-/-}$ male PBS

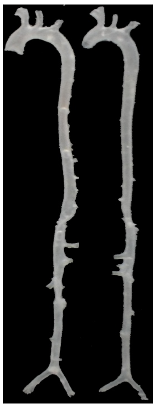

ApoE$^{-/-}$/ALOX15$^{-/-}$ male PBS

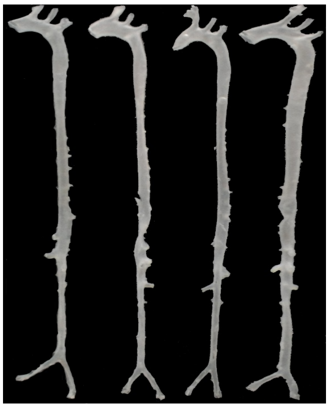

ApoE$^{-/-}$/ALOX12$^{-/-}$ male PBS

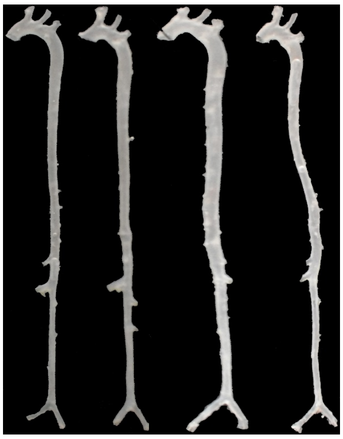

ApoE$^{-/-}$ female PBS

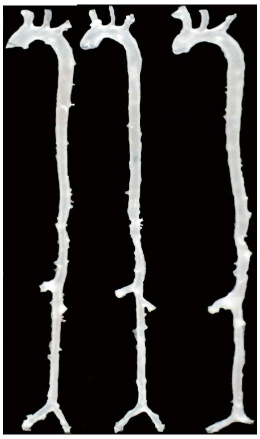

ApoE$^{-/-}$/ALOX15$^{-/-}$ female PBS

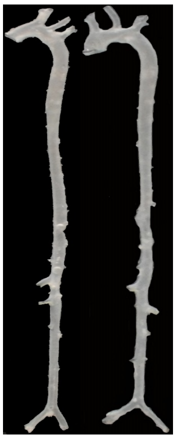

ApoE$^{-/-}$/ALOX12$^{-/-}$ female PBS

Supplementary Figure 6

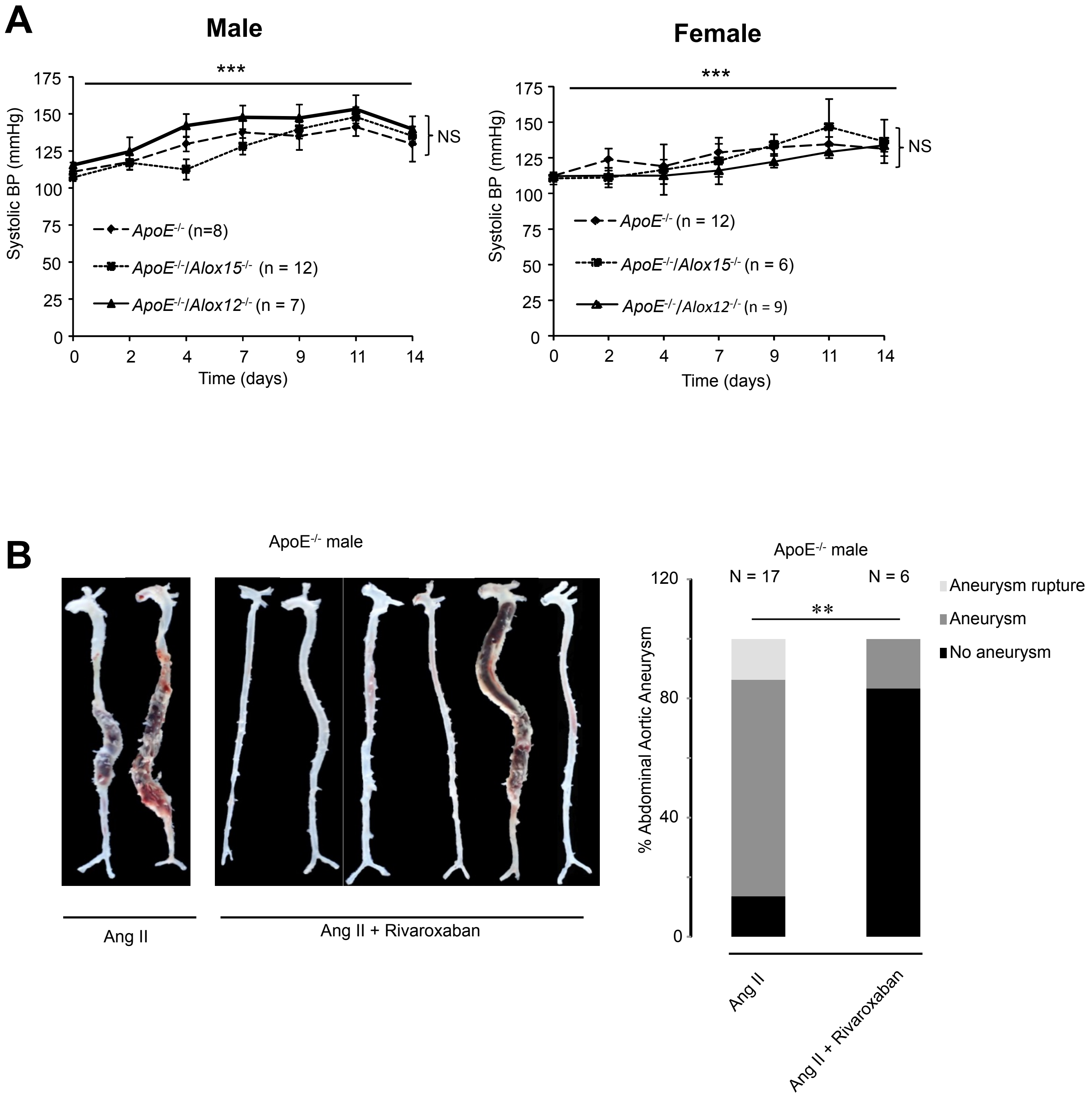

Supplementary Figure 7

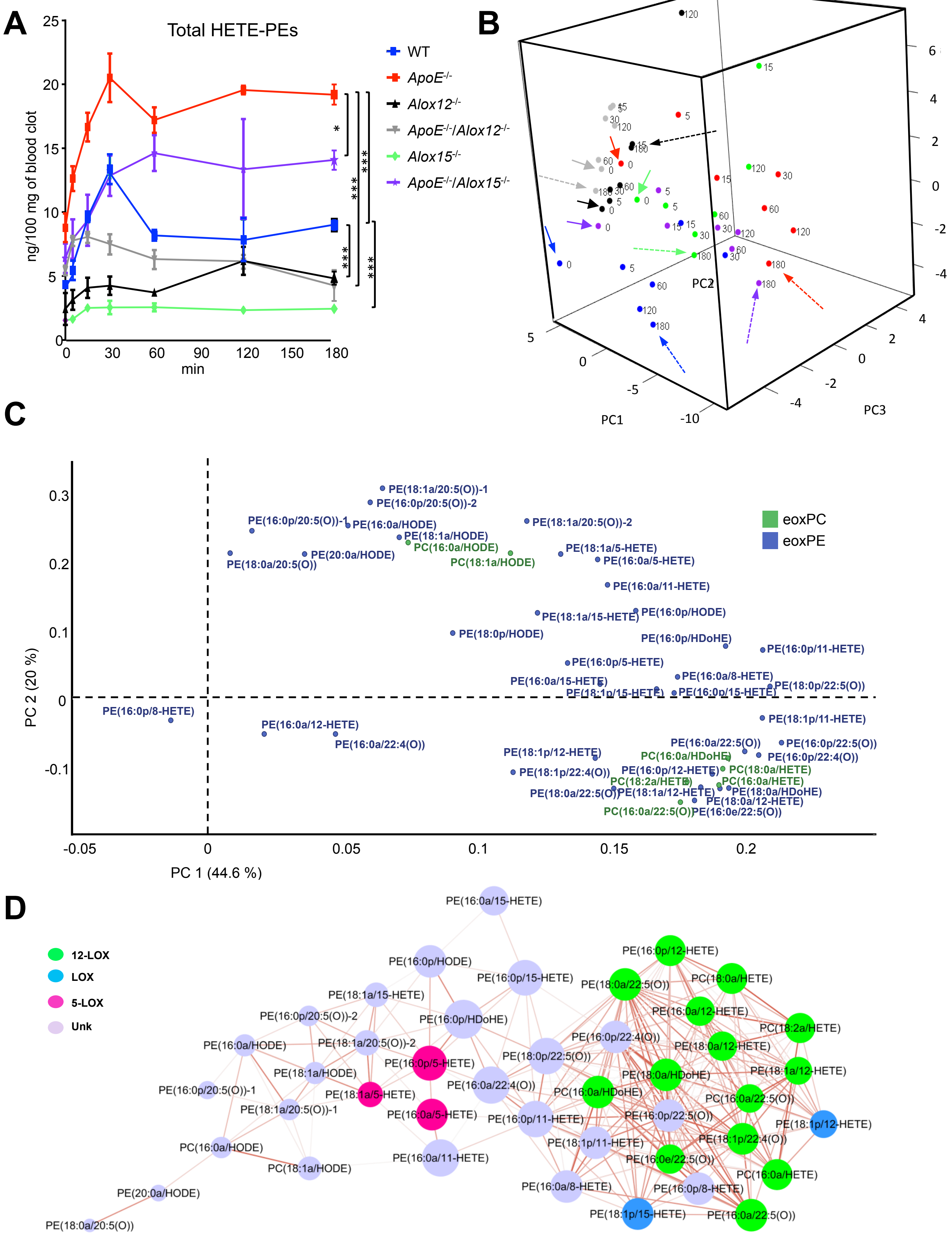

Supplementary Figure 8

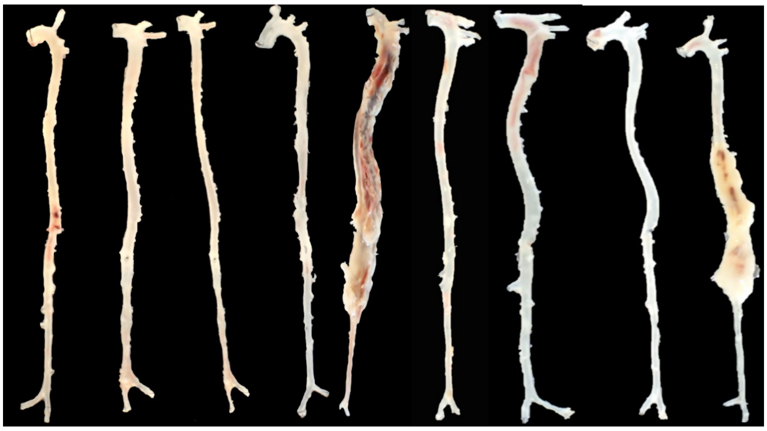

ApoE$^{-/-}$ with eoxPL

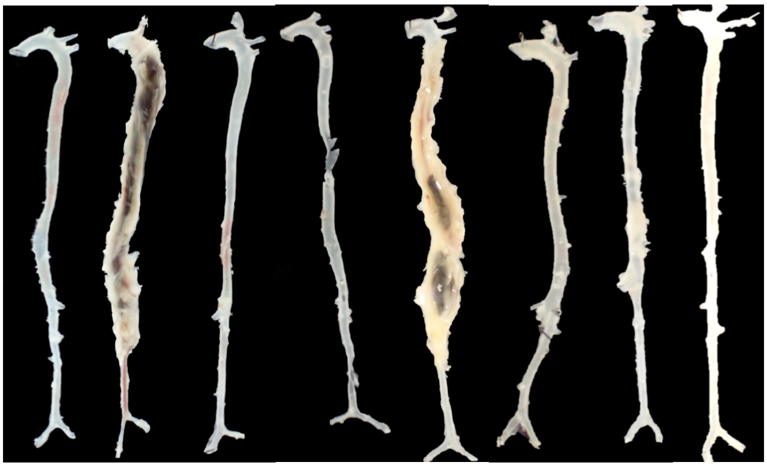

ApoE$^{-/-}$ with aPL

Supplementary Figure 9

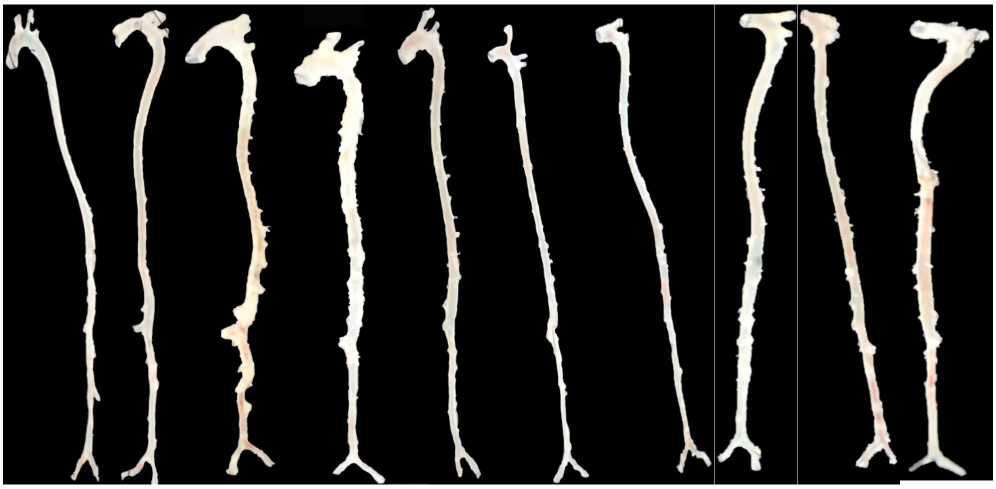

ApoE$^{-/-}$/*Alox15*$^{-/-}$ with eoxPL

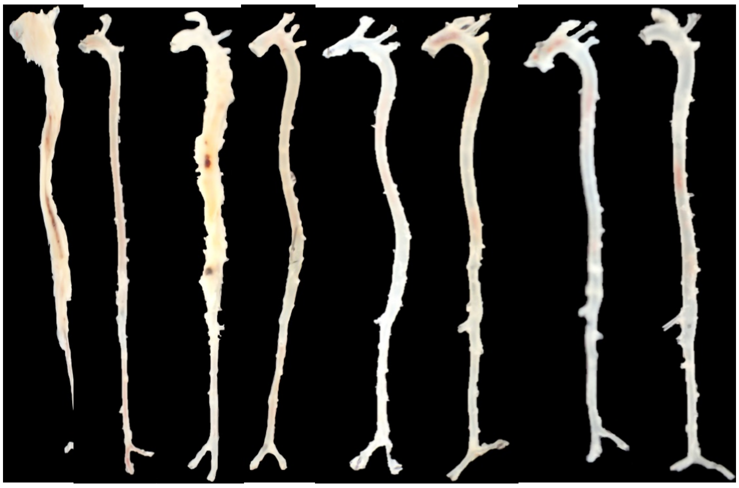

ApoE$^{-/-}$/*Alox15*$^{-/-}$ with aPL

Supplementary Figure 10

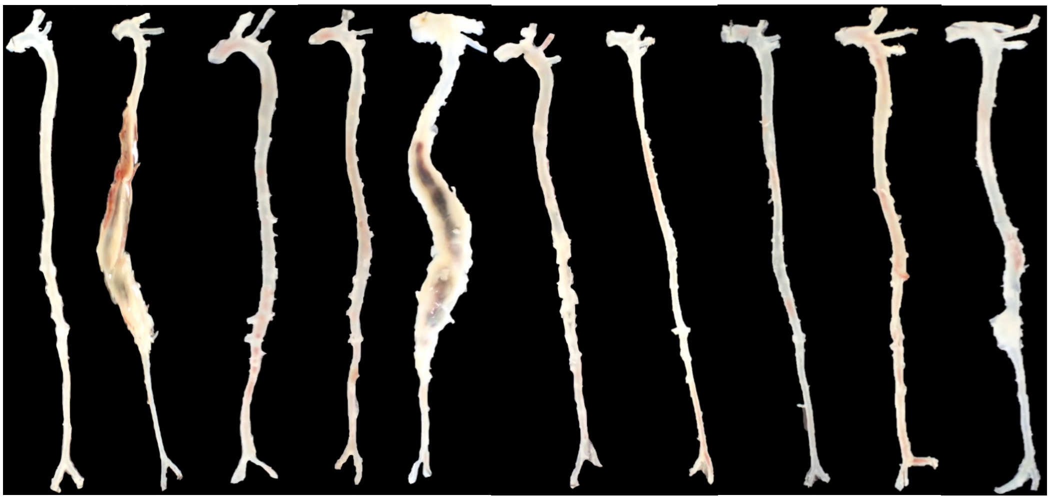

ApoE$^{-/-}$/*Alox12*$^{-/-}$ with eoxPL

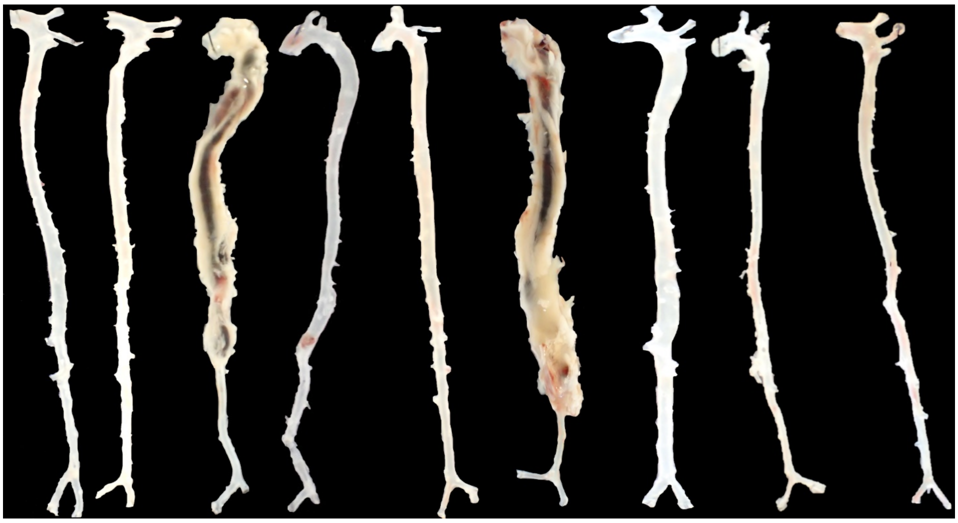

ApoE$^{-/-}$/*Alox12*$^{-/-}$ with aPL

Clarification: For this group which is summarized in **Figure** 6C, we processed n=10 samples, however only 9 aortae are shown above. During tissue processing, one of the samples was accidentally cut in half thus a photo record was not possible at that time, however the AAA outcome was recorded and has been included in the bar chart.
